# Myeloid loss of scaffolding protein menin promotes liver fibrosis *via* H3K36me3 reprogramming

**DOI:** 10.1016/j.jbc.2025.110471

**Published:** 2025-07-10

**Authors:** Qing Han, Yujun Chen, Junbo Yuan, Li Zhang, Qifan Zheng, Guanghui Jin

**Affiliations:** Department of Basic Medical Sciences, School of Medicine, Xiamen University; Xiamen, China

**Keywords:** *Men1*, SETD2, H3K36me3, liver fibrosis, epigenetics, macrophage

## Abstract

Menin, encoded by the *Men1* gene, is a scaffold protein broadly involved in regulating the cell phenotype through multiple histone modifications. Here, we discuss how menin contributes to liver macrophage (MAC) and hepatic stellate cell (HSC) fate determination, placing this contribution in the context of liver fibrosis pathogenesis. We revealed that *Men1* loss promoted CCL4- or high-fat diet-induced liver fibrosis. Menin regulated liver fibrogenesis primarily by modulating the activation of Kupffer cells (KCs)/HSCs rather than hepatocytes. The myeloid cell-specific knockout of *Setd2* but not *Kmt2a* mimicked the phenotype of *Men1* deletion. Menin/SETD2 suppressed the regeneration and activation of KCs by regulating IL-10 pathway activity through H3K36me3. In addition, the menin/SETD2/PPARγ complex coregulated SMAD7 expression through H3K36me3 in HSCs. JIB-04, an H3K36me3 agonist, effectively suppressed KC activation induced by *Men1* or *Setd2* deletion by reactivating IL-10 expression and further alleviated CCL4-induced liver fibrosis symptoms. Our results provide an interesting proof-of-concept for the therapeutic targeting of H3K36me3 remodeling to block liver fibrosis.

Advanced liver fibrosis is a major cause of liver disease-related deaths and can further progress to liver cancer. Therefore, elucidating the mechanisms underlying liver fibrosis will provide a strong theoretical basis for its treatment.

Liver fibrosis results from hepatocyte injury caused by carcinogens, high-fat diets, and alcohol, among other factors. The hepatocyte damage activates MACs to release inflammatory cytokines, which ultimately stimulate HSCs transdifferentiated into myofibroblasts to produce excess extracellular matrix (ECM), leading to stromal fibrosis in the liver (1, 2). As mediators of inflammatory responses, MACs play a pivotal role in the progression of hepatic fibrosis. Under physiological conditions, MACs in the liver are mainly KCs of embryonic origin (3). Hepatic injury signals activate KCs to release inflammatory factors/chemokines, such as IL-1β, IL-6, TNF-α, and CCL2. These factors recruit monocytes, which differentiate into MACs and, together with liver-resident KCs, activate liver inflammation (4). IL-10 inhibits LPS-induced glucose uptake and glycolysis to exert anti-inflammatory effects. A subgroup of MARCO^+^ KCs secretes IL-10 and other anti-inflammatory cytokines, suppressing neutrophil (NEU) aggregation and adhesion to regulate liver immune homeostasis (5, 6). Notably, the number of MARCO^+^ KCs is significantly reduced under pathological conditions such as nonalcoholic steatohepatitis (6). The essential role of the TGF-β/SMAD pathway in liver fibrosis has been demonstrated. TGF-β binds to its receptor, TGFβR, and induces the phosphorylation and nuclear translocation of downstream SMAD2/3, thereby increasing the expression of COL1A1 and other collagen-related genes (7). PPARγ is highly expressed in quiescent HSCs and inhibits liver fibrosis by negatively regulating the TGF-β/SMAD pathway (8). SMAD7 serves as a competitive inhibitor of TGFβR, suppressing this pathway activation and fibrosis progression, making it an important negative regulator of liver fibrosis (9). At present, there is still a lack of targeted therapeutic strategies based on the pathogenesis of liver fibrosis.

Chromatin remodeling is crucial for maintaining cell fate. Recent studies highlight the potential importance of histone remodeling in liver fibrosis. The histone methyltransferase MLL4 enhances NF-κB signaling through histone H3 lysine four methylation (H3K4me1) remodeling and promotes nonalcoholic steatohepatitis (10). The disruption of *Ezh1*/*2* in the liver upregulates the expression of *p14*^*ARF*^ through suppressing H3K27me3, leading to the premature differentiation of hepatocytes in the peripartum period and ultimately promoting liver fibrosis (11). Owing to the plasticity of chromatin reprogramming, an in-depth understanding of the epigenetic mechanisms of liver fibrosis could pave the way for targeted chromatin reprogramming treatment. Menin, encoded by the *Men1* gene, is a scaffold protein broadly involved in regulating the cell phenotype through multiple histone modifications (12). For example, menin interacts with MLL (*Kmt2a* gene) to maintain *Hoxa* expression *via* H3K4me3 (12). In acute leukemias of various phenotypes, the menin–MLL interaction recruits MLL-AF/DOT1L to *Hox* gene loci to drive oncogenesis (13). Recently, we demonstrated that menin is a key scaffold protein for SETD2-catalyzed chromatin H3K36me3. Menin mediates the site-specific chromatin recruitment of SETD2, maintaining CSF2 expression and alveolar macrophage (AM) homeostasis (14). The pathological importance of menin in liver diseases has rarely been studied. Differential expression profile analysis revealed that *Men1* expression was significantly upregulated in hepatocellular carcinoma (HCC) and was at a central node (15). We demonstrated that menin promotes HCC tumorigenesis and that the upregulation of menin expression in hepatocytes was negatively associated with prognosis (16, 17). These findings suggest potential pathological roles of menin in liver disease, although the mechanisms remain unclear.

In this study, we employed multiple genetically engineered mouse models to explore menin’s pathological roles, mechanistic contributions, and epigenetic features in liver fibrosis as well as to identify potential therapeutic targets.

## Results

### Loss of *Men1* promotes liver fibrosis

**Figure 1:**
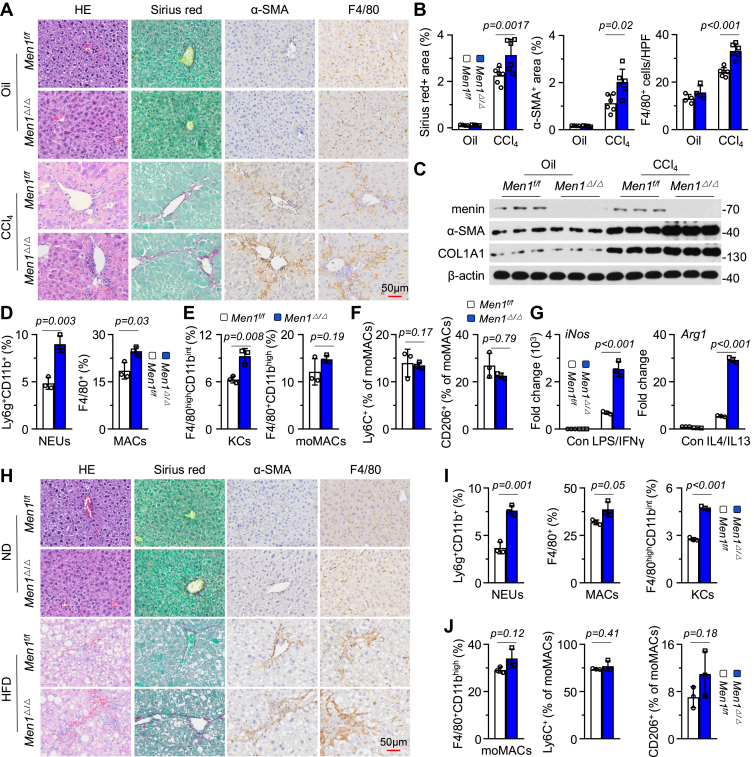
**Loss of *Men1* promotes liver fibrosis.***A*, representative staining of livers with HE, α-SMA, Sirius Red (SR), and F4/80 for *Men1*^*f/f*^ and *Men1*^*Δ/Δ*^ mice treated with 2 ml/kg oil or CCL4 *i.p.*, twice a week for 4 weeks (Scale bar = 50 μm). *B*, quantification of α-SMA, SR, and F4/80 staining of 1A (Oil n = 4, CCL4 n = 6). *C*, Western blot analysis of indicated protein expression in livers from above-mentioned models in 1A. *D* and *E*, nonparenchymal cells (NPCs) were isolated from livers of *Men1*^*f/f*^ and *Men1*^*Δ/Δ*^ mice treated with 2 ml/kg CCL4 *i.p.*, twice a week for 4 weeks (n = 3). Flow cytometry (FCM) was used to determine the proportion of Ly6g^+^CD11b^+^ neutrophils (NEUs), F4/80^+^ macrophages (MACs), F4/80^high^CD11b^int^ Kupffer cells (KCs), and F4/80^+^CD11b^high^ monocyte-derived macrophages (moMACs). *F*, FCM analysis the proportion of Ly6C^+^ and CD206^+^ in moMACs from above-mentioned models in 1D (n = 3). *G*, the KCs isolated from *Men1*^*f/f*^ and *Men1*^*Δ/Δ*^ mice were treated with LPS/IFN-γ or IL-4/IL-13, respectively, and the mRNA expression of *iNos* and *Arg1* was detected by RT-qPCR (n = 3). *H*, representative staining with HE, α-SMA, SR, and F4/80 in livers of *Men1*^*f/f*^ and *Men1*^*Δ/Δ*^ mice fed with a normal diet (ND) or high-fat diet (HFD) for 4 months (Scale bar = 50 μm). *I* and *J*, NPCs were isolated from livers of *Men1*^*f/f*^ and *Men1*^*Δ/Δ*^ mice fed with HFD for 4 months. FCM analysis of the proportion of NEUs, MACs, KCs, and moMACs and the proportion of Ly6C^+^ and CD206^+^ in moMACs (n = 3). Unpaired *t* test. Data are presented as mean ± SEM. Individual data points as independent biological replicates.

Wild-type (WT) and conditional UBC-Cre; *Men1*^*f/f*^ (*Men1*^*Δ/Δ*^) allele homozygous knockout (*KO*) mice were treated with CCL4 to establish liver fibrosis models (Fig. S1*A*). After 4 weeks, the serum alanine aminotransferase (ALT), aspartate aminotransferase (AST) levels, and liver/body weight ratio in the *Men1*^*Δ/Δ*^ group were significantly greater than those in the WT group (Fig. S1*B*). A uniform distribution of nodules on the liver surface was detected in the CCL4-treated group (Fig. S1*C*). HE staining revealed that the fibrous septum along the portal area was more obvious in the *Men1*^*Δ/Δ*^ group, with obvious inflammatory cell infiltration and hepatocyte necrosis areas (Fig. 1*A*). Stronger α-Smooth Muscle Actin (α-SMA), Sirius red (SR), and F4/80 staining was observed in the *Men1*^*Δ/Δ*^ group (Fig. 1, *A* and *B*). Western blot analysis revealed that *Men1*^*Δ/Δ*^ increased the expression of α-SMA and COL1A1 in the liver (Figs. 1*C* and S1*E*), accompanied by elevated mRNA levels of *α-Sma* and *Collagen1α1 (Colla1α1)* (Fig. S1*D*). There was no significant difference in TUNEL staining between the WT and *Men1*^*Δ/Δ*^ mice, suggesting that *Men1*^*Δ/Δ*^ did not affect hepatocyte apoptosis (Fig. S1, *F* and *G*). Considering the pivotal role of MAC activation in liver fibrosis, we explored the potential regulatory effects of menin on subsets of MACs. Flow cytometry (FCM) analysis revealed a significant increase in the proportion of Ly6g^+^CD11b^+^ NEUs and F4/80^+^ MACs in the *Men1*^*Δ/Δ*^ group (Fig. 1*D* and S1*H*). *Men1*^*Δ/Δ*^ increased the number of F4/80^high^CD11b^int^ KCs but had no significant effect on F4/80^+^CD11b^high^ monocyte-derived macrophages (moMACs) (18) (Figs. 1*E* and S1*I*), suggesting that menin predominantly impacts the regeneration of KCs rather than the recruitment of moMACs during the progression of liver fibrosis. Ly6C^+^ and CD206^+^ MACs have been reported to contribute to inflammatory responses during liver fibrosis (18, 19). Here, we did not observe a significant difference in the number of Ly6C^+^ and CD206^+^ moMACs subsets between the WT and the *Men1*^*Δ/Δ*^ groups (Figs. 1*F* and S1*I*). *Men1*^*Δ/Δ*^ significantly increased the mRNA expression of *Inducible Nitric Oxide Synthase (iNos)* and *Arginase 1* (*Arg1*), which are M1/M2 markers, in KCs (Fig. 1*G*).

We also established a high-fat diet (HFD)-induced liver fibrosis model (Fig. S1*J*). *Men1*^*Δ/Δ*^ also promoted HFD-induced liver fibrosis, as evidenced by enhanced α-SMA, SR, and F4/80 staining (Figs. 1*H* and S1*J*). The serum ALT and AST levels in the *Men1*^*Δ/Δ*^ group was significantly higher than those in the WT group (Fig. S1*K*). However, *Men1*^*Δ/Δ*^ did not affect the serum levels of triglyceride (TG) and total cholesterol (TC) (Fig. S1*K*). *Men1*^Δ/Δ^ increased the number of Ly6g^+^CD11b^+^ NEUs, F4/80^+^ MACs, and F4/80^high^CD11b^int^ KCs (Fig. 1*I*). *Men1*^*Δ/Δ*^ did not significantly affect the number and the polarization of F4/80^+^CD11b^high^ moMACs (Fig. 1*J*). Furthermore, among the organs examined, the kidneys presented the most pronounced changes. *Men1*^*Δ/Δ*^ obviously promoted HFD-induced renal fibrosis, as indicated by enhanced Masson staining and F4/80^+^ MAC infiltration (Fig. S1*L*). These results suggest that *Men1* deficiency promotes liver fibrosis.

### Myeloid deletion of *Men1* promotes liver fibrosis

The development of liver fibrosis is related to dynamic phenotypic changes in hepatocytes, KCs, and HSCs (1, 2). We were interested in which cell types are involved in menin-regulated liver fibrosis. We established hepatocyte-specific *Men1*-*KO* mice (albumin-cre; *Men1*^*f/f*^, *Men1*^*ΔH/ΔH*^), and a liver fibrosis model was established by treating mice with CCL4 (Fig. S1*A*). There was no significant difference in the liver/body weight ratio, serum ALT, and AST between *Men1*^*ΔH/ΔH*^ and WT mice (Fig. S2*A*). *Men1*^*ΔH/ΔH*^ did not promote liver fibrosis, as evidenced by the absence of significant differences in α-SMA, SR, and F4/80 staining (Fig. S2, *B* and *C*). TUNEL staining revealed that *Men1*^*ΔH/ΔH*^ did not significantly affect apoptosis (Fig. S2*D*). These results suggest that the regulatory effect of menin in regulating liver fibrosis is independent of hepatocytes.

**Figure 2:**
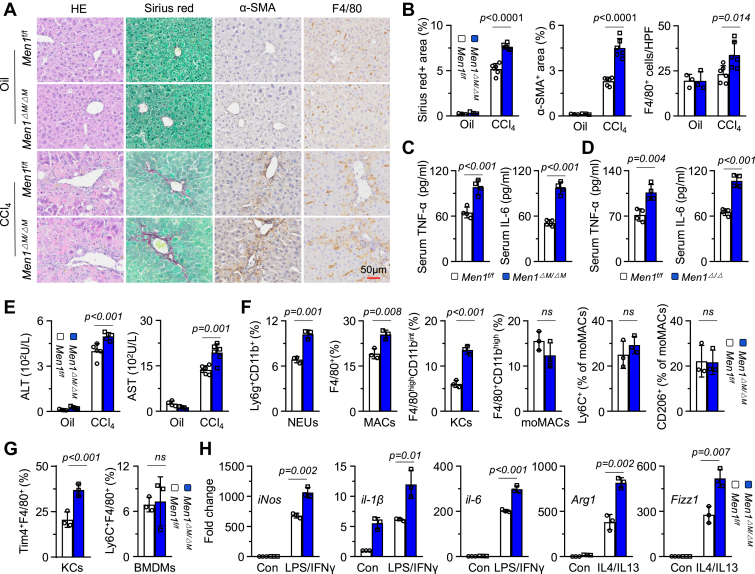
**Myeloid deletion of *Men1* promotes liver fibrosis.***A* and *B*, representative staining with HE, α-SMA, SR, and F4/80 in livers of *Men1*^*f/f*^ and *Men1*^*ΔM/ΔM*^ mice treated with 2 ml/kg oil or CCL4 *i.p.*, twice a week for 4 weeks (Scale bar = 50 μm). Quantification of α-SMA, SR, and F4/80 staining (Oil n = 3, CCL4 n = 6). *C*, *Men1*^*f/f*^ and *Men1*^*ΔM/ΔM*^ mice were treated with 2 ml/kg CCL4 *i.p.*, twice a week for 4 weeks. ELISA was used to detect the serum TNF-α and IL-6 levels (n = 4). *D*, *Men1*^*f/f*^ and *Men1*^*Δ/Δ*^ mice were treated with 2 ml/kg CCL4 *i.p.*, twice a week for 4 weeks. ELISA was used to detect the serum TNF-α and IL-6 levels (n = 4). *E*, quantification of serum ALT and AST levels of 2A (Oil n = 4, CCL4 n = 6). *F*, NPCs were isolated from liver of *Men1*^*f/f*^ and *Men1*^*ΔM/ΔM*^ mice treated with 2 ml/kg CCL4 *i.p.*, twice a week for 4 weeks (n = 3). FCM was used to determine the proportion of NEUs, MACs, KCs, and moMACs and the proportion of Ly6C^+^ and CD206^+^ in moMACs by using indicated antibodies. *G*, NPCs were isolated from liver of 6 to 8 weeks *Men1*^*f/f*^ and *Men1*^*ΔM/ΔM*^ mice, FCM was used to determine the proportion of Tim4^+^F4/80^+^ KCs (n = 3) (*left*); BMDMs were isolated from 6 to 8 weeks *Men1*^*f/f*^ and *Men1*^*ΔM/ΔM*^ mice, FCM was used to determine the proportion of Ly6C^+^F4/80^+^ BMDMs (n = 3) (*right*). *H*, the KCs isolated from *Men1*^*f/f*^ and *Men1*^*ΔM/ΔM*^ mice were treated with LPS/IFN-γ or IL-4/IL-13, respectively. The mRNA expressions of *iNos*, *il-1β*, *il-6*, *Arg1*, and *Fizz1* were detected by RT-qPCR (n = 3). Unpaired *t* test. Data are presented as mean ± SEM. Individual data points as independent biological replicates.

Next, we generated *Men1*^*f/f*^; *Lyz2-Cre*^+/−^ myeloid cell-specific *Men1*-*KO* mice (*Men1*^*ΔM/ΔM*^). In these mice, the sequence downstream of the *Lyz2-Cre*^+/−^ promoter is specifically deleted in myeloid-derived cells, including MACs, monocytes, and NEUs, and to a lesser extent in dendritic cells (14). Using a CCL4-induced liver fibrosis model, we found that we found that α-SMA and SR staining was enhanced and the number of F4/80^+^ MACs was also increased in the *Men1*^*ΔM/ΔM*^ group (Fig. 2, *A* and *B*). ELISAs revealed that the serum levels of TNF-α and IL-6 were significantly greater in the *Men1*^*ΔM/ΔM*^ group than in the WT (Fig. 2*C*), a finding that was consistent with observations in the *Men1*^*Δ/Δ*^ (Fig. 2*D*). Serum ALT and AST levels in the *Men1*^*ΔM/ΔM*^ group were also increased (Fig. 2*E*). *Men1*^*ΔM/ΔM*^ increased the protein expression of α-SMA and COL1A and the mRNA expression of *α-Sma* and *Colla1a1* in the liver (Fig. S2, *E* and *F*), which is in accordance with findings from isolated HSCs *in vivo* (Fig. S2*G*). *Men1*^*ΔM/ΔM*^ significantly increased the number of Ly6g^+^CD11b^+^ NEUs and F4/80^+^ MACs (Fig. 2*F*). *Men1*^*ΔM/ΔM*^ also increased the number of F4/80^high^CD11b^int^ KCs, whereas the number of F4/80^+^CD11b^high^ moMACs and their Ly6C^+^ and CD206^+^ subsets remained unaffected (Fig. 2*F*). Consistent with this, *Men1*^*ΔM/ΔM*^ increased the number of Tim4^+^F4/80^+^ KCs in normal liver without affecting the number of Ly6C^+^F4/80^+^ bone marrow-derived MACs (20) (Figs. 2*G* and S2*H*). *Men1*^*ΔM/ΔM*^ clearly promoted the mRNA and the protein expression of iNos, il-1β, il-6, Arg1, and Found in Inflammatory Zone 1 (Fizz1) (Figs. 2*H* and S2*I*). According to these results, we hypothesized that myeloid *Men1* depletion induces spontaneous liver fibrosis. After 8 months of normal diet feeding, HE staining results showed that the liver tissue structure was basically normal in *Men1*^*ΔM/ΔM*^ and WT groups, but the IHC results revealed that SR, α-SMA staining, and F4/80^+^ MAC infiltration were increased in the *Men1*^*ΔM/ΔM*^ group (Fig. S2*J*), suggesting that *Men1*^*ΔM/ΔM*^ results in a propensity for spontaneous liver fibrosis. These results indicate that *Men1* deficiency promotes liver fibrosis by activating KCs but not hepatocytes.

### Menin-mediated regulation of KCs depends on SETD2

**Figure 3:**
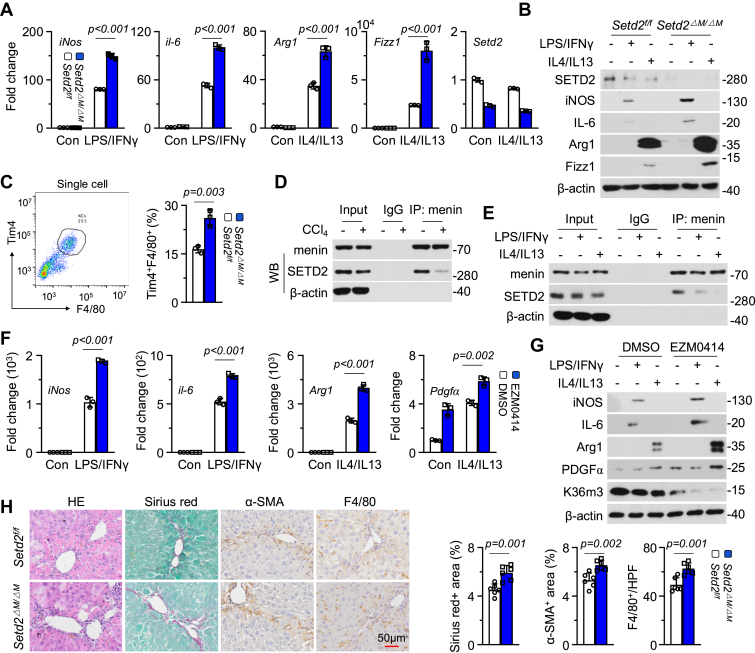
**Menin-mediated regulation of KCs depends on SETD2.***A* and *B*, the KCs isolated from *Setd2*^*f/f*^ and *Setd2*^*ΔM/ΔM*^ mice were treated with LPS/IFNγ or IL4/IL13, respectively. RT-qPCR analysis of *iNos*, *il-6*, *Arg1*, and *Fizz1* mRNA expression (n = 3) and western blotting detection of SETD2, iNOS, IL-6, Arg1, and Fizz1 protein expression. *C*, NPCs were isolated from livers of 6 to 8 weeks *Setd2*^*f/f*^ and *Setd2*^*ΔM/ΔM*^ mice, FCM was used to determine the proportion of Tim4^+^F4/80^+^ KCs (n = 3). *D*, the WT-C57BL/6J mice were treated with 2 ml/kg oil or CCL4 *i.p.*, twice a week for 3 weeks. MACs were isolated from these mice. Endogenous IP was performed with menin antibody and Western blot was used to detect the SETD2 and menin. *E*, the primary isolated KCs from 6 to 8 weeks WT-C57BL/6J mice were treated with LPS/IFNγ or IL4/IL13, respectively. Endogenous IP was performed with menin antibody and Western blot was used to detect SETD2 and menin. *F* and *G*, the primary isolated KCs from 6 to 8 weeks WT-C57BL/6J mice were treated with LPS/IFNγ or IL4/IL13 and simultaneously administered EZM0414 (5 μM) for 48 h, respectively. RT-qPCR was used to detect *iNos*, *il-6*, *Arg1*, and *Pdgfα* mRNA expression (n = 3). Western blot was used to detect iNOS, IL-6, Arg1, PDGFα, and H3K36me3. *H*, representative and quantification of HE, SR, α-SMA, and F4/80 staining in livers of *Setd2*^*f/f*^ and *Setd2*^*ΔM/ΔM*^ mice treated with 2 ml/kg CCL4 *i.p.*, twice a week for 4 weeks (n = 6) (Scale bar = 50 μm). Unpaired *t* test. Data are presented as mean ± SEM. Individual data points as independent biological replicates.

We investigated the epigenetic properties of menin in regulating liver MACs. *Men1*-*KO* caused an obvious decrease in the overall level of H3K36me3 but not H3K4me3, H3K9me3, or H3K27me3 in KCs (Fig. S3*A*). SETD2 specifically tri-methylates Lys-36 of histone H3 using demethylated Lys-36 as a substrate, and the methylation of this residue is associated with active chromatin (21). We established a myeloid *Setd2*-*KO* animal model (lyz2-cre; *Setd2*^*f/f*^, *Setd2*^*ΔM/ΔM*^). *Setd2*^*ΔM/ΔM*^ significantly promoted the mRNA and protein expression of iNos, il-6, Arg1, and Fizz1 (Figs. 3, *A* and *B* and S3*B*). FCM analysis revealed that *Setd2*^*ΔM/ΔM*^ also increased the number of Tim4^+^F4/80^+^ KCs in normal liver (Fig. 3*C*). IP revealed that in liver MACs, menin interacted with SETD2 under basal conditions and this interaction was attenuated by exposure to CCL4 (Fig. 3*D*). Exposure to either LPS/IFNγ or IL4/IL13 also inhibited the menin/SETD2 interaction in KCs (Fig. 3*E*). Exposure to EZM0414, a specific inhibitor of H3K36me3 (22), promoted the mRNA and protein expression of iNos, il-6, Arg1, and Pdgfα (Figs. 3, *F* and *G* and S3*C*). Using a CCL4-induced liver fibrosis model, *Setd2*^*ΔM/ΔM*^ led to an increase in SR, α-SMA, and F4/80 staining (Fig. 3*H*). However, *Kmt2a*^*ΔM/ΔM*^ did not regularly increase the mRNA expression of *iNos*, *il-1β*, *Arg1*, and *Fizz1* or the protein expression of iNOS and Arg1 (Fig. S3, *D–F*). *Kmt2a*^*ΔM/ΔM*^ also did not affect the number of Tim4^+^F4/80^+^ KCs in normal liver (Fig. S3*G*). Exposure to MI-3, which specifically disrupts the menin/MLL interaction (23), slightly promoted *iNos* expression but significantly inhibited *il-6*, *Arg1*, and *Fizz1* expression (Fig. S3, *H* and *I*). These results suggest that the menin-regulated KCs phenotype is dependent on SETD2-mediated H3K36me3 but not MLL-mediated H3K4me3.

### Menin/SETD2 controls KC remodeling through IL-10

**Figure 4:**
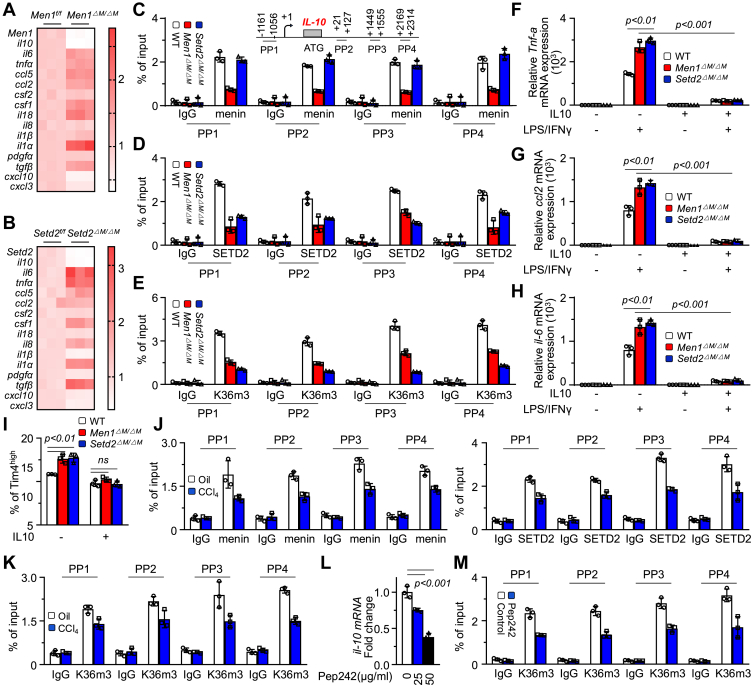
**Menin/SETD2 controls KC remodeling through IL-10.***A* and *B*, RT-qPCR analysis of indicated genes in KCs isolated from 6 to 8 weeks *Men1*^*f/f*^ and *Men1*^*ΔM/ΔM*^, *Setd2*^*f/f*^ and *Setd2*^*ΔM/ΔM*^ mice (n = 3). *C–E*, diagrams showing the primer pairs (PPs) designed for ChIP targeting the regulatory region of *il-10*. ChIP-qPCR analysis of enrichment of menin, SETD2, and H3K36me3 on *il-10* regulatory region in KCs isolated from WT, *Men1*^*ΔM/ΔM*^ and *Setd2*^*ΔM/ΔM*^ mice (n = 3). *F–H*, the KCs isolated from WT, *Men1*^*ΔM/ΔM*^ and *Setd2*^*ΔM/ΔM*^ mice were treated with LPS/IFNγ and simultaneously treated with IL-10 (50 ng/ml) for 48 h. RT-qPCR analysis of *Tnf-a*, *ccl2*, and *il-6* mRNA expression (n = 3). *I*, the KCs isolated from WT, *Men1*^*ΔM/ΔM*^ and *Setd2*^*ΔM/ΔM*^ mice were treated with IL-10 (50 ng/ml) for 48 h. FCM was used to determine the proportion of Tim4^high^ KC subsets (n = 3). *J* and *K*, ChIP-qPCR analysis of enrichment of menin, SETD2, and H3K36me3 on *il-10* regulatory region in KCs isolated from 6 to 8 weeks WT-C57BL/6J mice treated with 2 ml/kg oil or CCL4 *i.p.*, twice a week for 3 weeks (n = 3). *L*, the KCs isolated from 6 to 8 weeks WT-C57BL/6J mice were treated with Pep242 for 48 h, and RT-qPCR was used to detect *il-10* expression (n = 3). *M*, the KCs isolated from 6 to 8 weeks WT-C57BL/6J mice were treated with Pep242 (50 μg/ml) for 48 h, ChIP-qPCR was used to detect the enrichment of H3K36me3 at *il-10* gene regulatory region (n = 3). Unpaired *t* test. Data are presented as mean ± SEM. Individual data points as independent biological replicates.

We explored the major genes regulated by menin/SETD2 in liver MACs. We screened cytokines/chemokines associated with the differentiation/activation of MACs (24). Both *Men1*^*ΔM/ΔM*^ and *Setd2*^*ΔM/ΔM*^ significantly inhibited the expression of the *il-10* and *cxcl10* and promoted the expression of *il-6*, *tnf-α*, *ccl5*, *ccl2*, *csf1*, *il-18*, and *il-1α* (Fig. 4, *A* and *B*). Since H3K36me3 is associated with active chromatin, we hypothesized that the upregulation of target genes by *Men1*^*ΔM/ΔM*^/*Setd2*^*ΔM/ΔM*^ might be due to a negative regulatory mechanism. Different from KCs, *Men1 KO* significantly inhibited the transcription of *csf2*, *il-8*, *il-1β*, and *il-1α* in AMs (Fig. S4*A*). IL-10, which is secreted by Marco^+^ KCs, is a key anti-inflammatory cytokine for liver fibrosis (6). Exposure to EZM0414 inhibited *il-10* mRNA expression in a dose-dependent manner (Fig. S4*B*). ChIP assays confirmed that menin and SETD2 clearly bound to the *il-10* regulatory region, accompanied by abundant H3K36me3 modifications under basal conditions (Fig. 4, *C–E*). *Men1*^*ΔM/ΔM*^ clearly inhibited the enrichment of menin, SETD2, and H3K36me3 at the *il-10* regulatory region, whereas *Setd2*-*KO* suppressed the enrichment of SETD2 and H3K36me3 but not menin (Fig. 4, *C–E*). These results suggest that menin recruits SETD2 to specific chromatin loci for H3K36me3 catalysis. However, the enrichment of H3K4me3 was detected only at promoter sites (PP1 and PP2) and was not affected by *Men1*^*ΔM/ΔM*^ (Fig. S4*C*). Moreover, *Kmt2a*^*ΔM/ΔM*^ did not inhibit the mRNA expression of *il-10* in KCs but rather promoted it (Fig. S4*D*).

The exposure of recombinant IL-10 (rIL-10) clearly suppressed the transcription of the *Tnf-α*, *ccl2*, *il-6*, and *iNos* genes promoted by *Men1*^*ΔM/ΔM*^ or *Setd2*^*ΔM/ΔM*^ (Figs. 4, *F–H* and S4*E*). The number of Tim4^high^ KCs was increased by *Men1*^*ΔM/ΔM*^ or *Setd2*^*ΔM/ΔM*^ but inhibited by exposure to rIL-10 (Figs. 4*I* and S4*F*). Exposure to CCL4 obviously reduced the enrichment of menin, SETD2, and H3K36me3 at the *il-10* gene regulatory region (Fig. 4, *J* and *K*). We demonstrated that menin-regulated CSF2/IL-6 is critical for AM development/activation (14). Although menin binds to the *Csf2* and *il-6* promoter sites in KCs, no substantial SETD2 or H3K36me3 enrichment was observed (Fig. S4*G*). These results indicate different epigenetic properties of menin in AMs and KCs. The peptide pep242, which specifically inhibits the interaction of menin/SETD2 (14) (Fig. S4*H*), dose-dependently inhibited the mRNA expression of *il-10* and suppressed *il-10* gene regulatory region-associated H3K36me3 enrichment in KCs (Fig. 4, *L* and *M*). These results suggest that the maintenance of IL-10 by menin/SETD2 through H3K36me3 is critical for the phenotypic regulation of liver MACs.

### Menin/SETD2 collectively controls HSC activation

**Figure 5:**
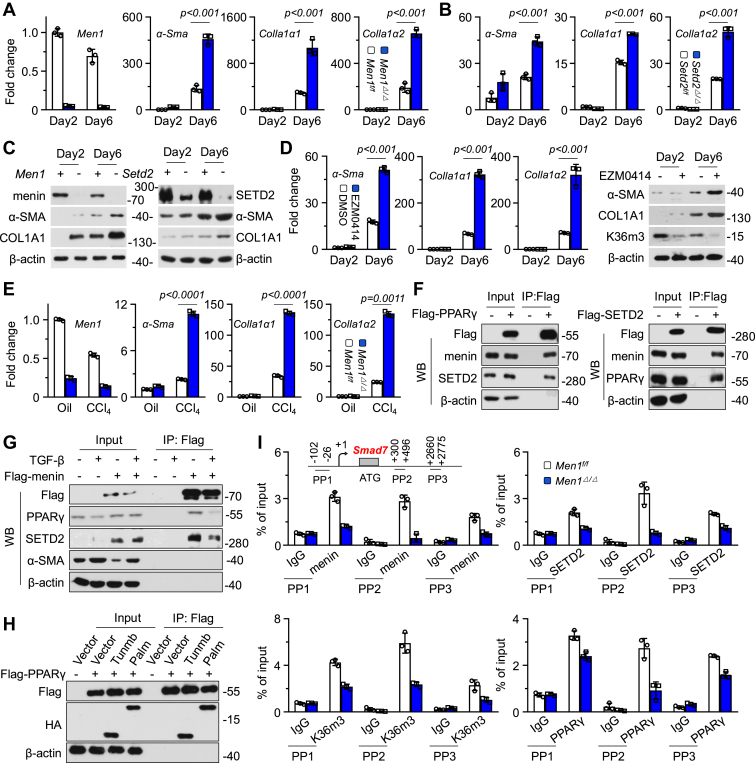
**Menin/SETD2 collectively controls HSC activation.***A*, the HSCs isolated from *Men1*^*f/f*^ and *Men1*^*Δ/Δ*^ mice were cultured in *vitro* for two or 6 days (n = 3). RT-qPCR analysis of *Men1*, *α-Sma*, *Colla1α1*, and *Colla1α2* mRNA expression. *B*, the HSCs isolated from *Setd2*^*f/f*^ and S*etd2*^*Δ/Δ*^ mice were cultured *in vitro* for two or 6 days (n = 3). RT-qPCR analysis of *α-Sma*, *Colla1α1*, and *Colla1α2* mRNA expression. *C*, the HSCs isolated from *Men1*^*f/f*^ and *Men1*^*Δ/Δ*^, *Setd2*^*f/f*^ and S*etd2*^*Δ/Δ*^ mice were cultured in *vitro* for two or 6 days. Western blot analysis of menin/SETD2, α-SMA, and COL1A1 expression. *D*, the HSCs isolated from 6 to 8 weeks WT-C57BL/6J mice were cultured in *vitro* for two or 6 days and treated with EZM0414 (5 μM) (n = 3). RT-qPCR was used to detect α*-Sma*, *Colla1α1*, and *Colla1α2* expression (n = 3). Western blot was used to detect α-SMA, COL1A1, and H3K36me3. *E*, the HSCs were isolated from *Men1*^*f/f*^ and *Men1*^*Δ/Δ*^ mice treated with 2 ml/kg oil or CCL4 *i.p.*, twice a week for 4 weeks. RT-qPCR analysis of *Men1*, *α-Sma*, *Colla1α1*, and *Colla1α2* mRNA expression (n = 3). *F*, Co-IP was performed with anti-Flag antibody labeled magnetic beads in LX2 cells transfected with Flag-PPARγ or Flag-SETD2. Western blot was used to detect menin, SETD2, and PPARγ. *G*, Co-IP was performed with anti-Flag antibody labeled magnetic beads in LX2 cells transfected with Flag-menin and treated with TGF-β (10 ng/ml) for 48 h. Western blot was used to detect Flag, SETD2, PPARγ, and α-SMA. *H*, Co-IP was performed with anti-Flag antibody labeled magnetic beads in 293T cells transfected with Flag-PPARγ and HA-Thumb/HA-Palm. Western blot was used to detect Flag and HA. *I*, Diagrams showing the primer pairs (PPs) designed for ChIP targeting the regulatory region of *Smad7*. ChIP-qPCR analysis of enrichment of menin, SETD2, PPARγ, and H3K36me3 at the *Smad7* regulatory region in HSCs isolated from *Men1*^*f/f*^ and *Men1*^*Δ/Δ*^ mice (n = 3). Unpaired *t* test. Data are presented as mean ± SEM. Individual data points as independent biological replicates.

HSCs are the key effectors of liver fibrosis and promote liver fibrosis by the excessive secretion of ECM (1, 2). Primary isolated HSCs were spontaneously activated by *in vitro* culture (25). *Men1*^*Δ/Δ*^ or *Setd2*^*Δ/Δ*^ significantly promoted the mRNA and protein expression of fibrosis-related ECM genes such as α-Sma, Colla1α1, and Colla1α2 (Figs. 5, *A–C* and S5*A*). shRNA interference of *Men1* promoted the expression of α-SMA and COL1A1 in LX2 cells (Fig. S5, *B* and *C*). However, shRNA targeting *Men1* or *Setd2* had no significant effect on the proliferation of LX2 cells stimulated with TGF-β (Fig. S5*D*). Treatment with EZM0414 promoted the mRNA or protein expression of α-Sma, Colla1α1, and Colla1α2 (Fig. 5*D* and S5*E*). *Men1 KO* further promoted the CCL4-induced mRNA expression of *α-Sma*, *Colla1α1*, and *Colla1α2* of HSC *in vivo* (Fig. 5*E*). PPARγ activation is closely associated with the senescence and inactivation of HSCs (8, 26). It has also been reported that menin interacts with PPARγ in adipocytes (27). IP results confirmed an interaction among menin, SETD2, and PPARγ in LX2 cells under basal conditions (Fig. 5*F*), which was blocked by exposure to TGF-β (Fig. 5*G*). The menin protein is composed of four domains: NTD, Thumb, Palm, and Fingers (14) (Fig. S5*F*). IP results confirmed that menin interacted with PPARγ through the Palm and Fingers domains (Figs. 5*H* and S5*G*). Exposure to GW9662, a PPARγ inhibitor, activated HSCs, as evidenced by the upregulation of the mRNA and protein expression of α-Sma and Colla1α1 (Fig. S5, *H* and *I*). These results suggest that the menin-mediated regulation of HSC phenotypes possibly depends on SETD2 and PPARγ. SMAD7 inhibits the activation of HSCs, and the upregulation of SMAD7 expression suppresses the progression of liver fibrosis and HCC (9, 28). Exposure to GW9662 inhibited the expression of Smad7 at both the mRNA and protein levels (Fig. S5*J*), and *Men1*^*Δ/Δ*^ or *Setd2*^*Δ/Δ*^ also significantly inhibited Smad7 expression (Fig. S5, *K* and *L*). ChIP analysis clearly demonstrated that menin bound to the *Smad7* regulatory region, accompanied by the enrichment of SETD2, H3K36me3, and PPARγ under basal conditions, and that *Men1* KO inhibited this binding (Fig. 5*I*). Furthermore, exposure to CCL4 suppressed H3K36me3 enrichment at the *Smad7* regulatory region in HSCs (Fig. S5*M*). Pep242 blocked the interaction between menin and SETD2 and inhibited *Smad7* mRNA expression in a dose-dependent manner (Fig. S5*N*). These results suggest that menin/SETD2/PPARγ coregulate HSC activation through SMAD7.

### Therapeutic potential of targeting H3K36me3 reprogramming in liver fibrosis

**Figure 6:**
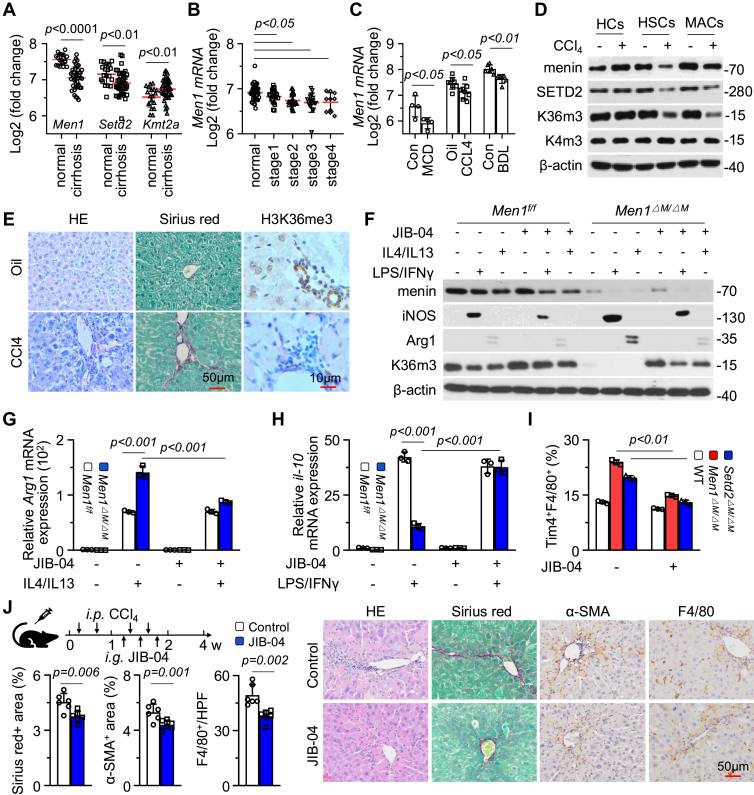
**Therapeutic potential of targeting H3K36me3 reprogramming in liver fibrosis.***A*, databases (GSE14323) analysis of *Men1*, *Setd2*, and *Kmt2a* mRNA expression in normal and cirrhotic livers. *B*, databases (GSE84044) analysis of *Men1* mRNA expression in different stages of liver fibrosis. *C*, databases analysis of *Men1* mRNA expression in mouse liver fibrosis models induced by MCD (GSE35961), CCL4 (GSE141821), or BDL (GSE152494). *D*, Western blot detection of indicated antibodies in hepatocytes (HCs), HSCs, MACs isolated from WT-C57BL/6J mice treated with 2 ml/kg oil or CCL4 *i.p.*, twice/week for 3 weeks. *E*, representative staining of liver tissues with HE, SR, and H3K36me3 for WT-C57BL/6J mice treated with 2 ml/kg oil or CCL4 *i.p.*, twice/week for 4 weeks (Scale bar = 50 μm). *F–H*, the KCs isolated from WT, *Men1*^*ΔM/ΔM*^ and *Setd2*^*ΔM/ΔM*^ mice were treated with LPS/IFNγ or IL4/IL13, respectively, and simultaneously treated with JIB-04 (5 μM) for 48 h. Western blot was used to detect iNOS, Arg1, and H3K36me3. RT-qPCR was used to detect *Arg1* and *il-10* mRNA expression (n = 3). *I*, NPCs were isolated from liver of WT-C57BL/6J mice treated with 40 mg/kg JIB-04 through intragastric gavage, three times a week for 3 weeks, FCM was used to determine the proportion of Tim4^+^F4/80^+^ KCs (n = 3). *J*, schematic of JIB-04 treatment in CCL4-induced liver fibrosis and quantification of α-SMA, SR, and F4/80 staining (*left*) (Control n = 6, JIB-04 n = 6). Representative staining of liver tissues with α-SMA, SR, and F4/80 for WT mice treated with 2 ml/kg CCL4 *i.p.*, twice/week for 3 weeks, therapeutically, treated with JIB-04 (40 mg/kg) through intragastric gavage, three times a week for 3 weeks (Scale bar = 50 μm) (*right*). Unpaired *t* test. Data are presented as mean ± SEM. Individual data points as independent biological replicates.

On the basis of the above observations, we evaluated the clinical relevance of H3K36me3 regulation by menin/SETD2 in liver fibrosis. An analysis of the clinical liver cirrhosis database revealed that the mRNA expression of *Men1* and *Setd2* in cirrhotic livers was significantly lower than that in normal livers, whereas the expression of *Kmt2a* was slightly greater (Fig. 6*A*). An analysis of different clinical stages of liver fibrosis revealed that *Men1* expression was negatively correlated with clinical stage, while there was no significant difference between stage 3 and stage 4 (Fig. 6*B*). *Men1* expression was significantly decreased in mouse liver fibrosis models induced by Methionine-Choline Deficient Diet (MCD), CCL4, and Bile Duct Ligation (BDL) (Fig. 6*C*). Moreover, *Men1* expression was negatively correlated with the expression of numerous fibrosis-related *collagen* genes (*r* < 0, *p* < 0.05), whereas *Kmt2a* expression was positively correlated (*r* > 0, *p* < 0.05) in clinical HCC samples (Fig. S6, *A* and *B*).

We isolated primary hepatocytes, MACs, and HSCs and found that exposure to CCL4 caused a reduction in menin, SETD2, and H3K36me3 but not H3K4me3 in HSCs and MACs (Figs. 6*D* and S6*C*). Exposure to CCL4 had no obvious effect on the expression of menin, SETD2, and H3K36me3 in hepatocytes (Figs. 6*D* and S6*C*). IHC revealed that H3K36me3 staining was obviously reduced in intrahepatic nonparenchymal cells around the portal area of liver tissue in the CCL4-induced fibrosis model (Fig. 6*E*). These results suggest that H3K36me3 remodeling occurs during liver fibrosis and that the reactivation of H3K36me3 may alleviate the symptoms of liver fibrosis. Exposure to JIB-04, an agonist of H3K36me3 (29), increased global H3K36me3 in chromatin and further inhibited the upregulation of iNOS and Arg1 expression induced by *Men1 KO* (Figs. 6, *F* and *G* and S6*D*). The inhibition of *il-10* mRNA expression by *Men1 KO* was markedly increased by exposure to JIB-04 (Fig. 6*H*). Consistent with these findings, exposure to JIB-04 clearly decreased the number of Tim4^+^F4/80^+^ KCs increased by *Men1* or *Setd2* KO (Fig. 6*I*). Furthermore, a CCL4-induced liver fibrosis model was established and treated with JIB-04 (Fig. 6*J*). Treatment with JIB-04 clearly alleviated the symptoms of liver fibrosis, as demonstrated by a decrease in SR and α-SMA staining and a decrease in the infiltration of F4/80^+^ MACs (Fig. 6*J*). These results suggest that H3K36me3 remodeling is a potential target for the treatment of liver fibrosis.

## Discussion

Although the importance of menin in multisystem diseases has been widely demonstrated, the potential pathological role of menin in liver diseases has not been fully investigated.

The present study clearly demonstrated the pathological importance of menin in liver fibrosis using a variety of models. Clinical database analysis also revealed that *Men1* expression was significantly associated with liver fibrosis. Herein, menin regulated liver fibrogenesis primarily by phenotypically modulating the activation of KCs/HSCs rather than hepatocytes. *Men1* deficiency enhanced the sensitivity of HSCs and KCs to external environmental pathogenic factors, promoted the expression of inflammatory factors and fibrosis-related collagen, and increased the risk of liver fibrosis. However, hepatocyte-specific *Men1* deficiency did not affect the risk of liver fibrosis. High menin expression in hepatocytes promoted HCC development, which was closely related to the degree of malignancy and size of HCC (15, 16, 17). These results suggest that the biological functions of menin in hepatocytes and KCs/HSCs are relatively separate, which may be related to the different epigenetic regulatory properties of menin. For example, menin promotes the activation of oncogenes such as *Yap1* mainly through MLL-mediated H3K4me3 in hepatocytes (17). However, menin regulates the transcription of *il-10* and *Smad7* mainly through SETD2-mediated H3K36me3 in KCs and HSCs. The *Setd2*^*ΔM/ΔM*^ mouse model mimicked the *Men1*^*ΔM/ΔM*^ phenotype well, indicating that menin/SETD2 functions as a complex to regulate liver MAC homeostasis. Neither the genetic nor pharmacological inhibition of MLL significantly affected the KC phenotype, suggesting that the molecular mechanism of the menin-regulated KC phenotype is separate from that of MLL-mediated H3K4me3.

This study confirmed the diversity and cellular specificity of the biological functions and epigenetic properties of menin. Menin is a major tumor suppressor in multiple endocrine neoplasia, and it plays an important role as an oncogene in MLL-AF fusion protein-induced leukemia (13, 30). Interestingly, although both are tissue-resident MACs, menin has completely opposite functions in KC and AM development. We hypothesize that this may be attributed to the different transcriptional properties of target genes regulated by menin in KCs and AMs. There are obvious differences between KCs and AMs in terms of embryonic origin and developmental regulatory mechanisms (31, 32). KCs originate from erythromyeloid precursors derived from the embryonic yolk sac (31), whereas AMs arise from monocytes of the fetal liver (32). CSF2 is a key lineage-determining factor for AM development and maturation. However, *Csf2 KO* did not significantly affect the number of KCs in the normal liver; KC proliferation and restoration were not affected by *Csf2 KO* after partial hepatectomy (33, 34). Menin/SETD2 maintained CSF2 expression through H3K36me3 in AMs, which is critical for AM development (14), whereas menin did not directly regulate CSF2 in KCs. Although menin/SETD2 collectively regulates MAC phenotypes and gene transcription in different tissues, the recruitment of SETD2 at specific gene promoter sites varies across different tissues. For example, menin maintains CSF2 expression by recruiting SETD2 to cause H3K36me3 in AMs (14). Although menin bound to the *csf2* promoter, it did not affect SETD2 recruitment or H3K36me3 modification in KCs (Fig. S5*G*). Herein, menin/SETD2 regulated the transcription of *il-10* and the KC phenotype through H3K36me3. However, AMs barely expressed *il-10*, and *Men1 KO* had no effect on *il-10* expression (Fig. S5*A*). Therefore, we hypothesize that the transcriptional regulatory properties of menin on AMs and KCs may be the molecular basis for the inconsistent phenotypic regulation. Whether this is related to the different microenvironments of the lung and liver requires further investigation. The cytological and histological basis of menin epigenetic properties, such as the context in which menin specifically interacts with MLL or SETD2, remains unclear.

Based on the importance of menin-mediated H3K36me3 reprogramming in the development of liver fibrosis, we explored the potential application of the targeted reversal of H3K36me3 in the treatment of liver fibrosis. Indeed, JIB-04, an H3K36me3 agonist, effectively suppressed KC activation by reactivating IL-10 expression and alleviated symptoms of CCL4-induced liver fibrosis. Our results provide an interesting proof-of-concept for the therapeutic targeting of H3K36me3 remodeling to inhibit the progression of liver fibrosis promoted by abnormal menin/SETD2 inactivation. In summary, the present study uncovered the pathological significance of menin/SETD2-mediated H3K36me3 reprogramming in liver fibrosis, providing new insights into the reversible intervention of liver fibrosis.

## Materials and methods

### Mice

The experimental protocols for *Men1*^*f/f*^ and *Kmt2a*^*f/f*^ mice have been previously described (35, 36). *Setd2*^*f/f*^ mice were purchased from GemPharmatech. Myeloid-specific knockout mice (*Men1*^*ΔM/ΔM*^, *Kmt2a*^*ΔM/ΔM*^, *Setd2*^*ΔM/ΔM*^) were generated by crossing with Lysozyme-Cre (Lyz2-Cre) mice from Jackson Laboratory. Whole-body knockout strains (*Men1*^*Δ/Δ*^, *Setd2*^*Δ/Δ*^) were developed by crossing with Ubiquitin-Cre (Ubc-Cre) mice and induced by tamoxifen for five consecutive days, with knockouts verified *via* PCR. Liver-specific knockout mice (*Men1*^*ΔH/ΔH*^) were generated by crossing *Men1*^*f/f*^ mice with Albumin-Cre (Alb-Cre) mice from Jackson Laboratory (37). Mice were housed in an SPF-grade environment and fed a standard diet at the Experimental Animal Center of Xiamen University. All animal experiments were approved by the Institutional Animal Care and Use Committee of Xiamen University (XMULAC20200161).

### Induction of liver fibrosis

Hepatotoxic Drug-Induced Fibrosis: Mice aged 8 to 10 weeks were intraperitoneally injected with 2 ml/kg of corn oil or CCL4 (diluted in a 1:9 ratio with corn oil) twice weekly for 4 weeks. For therapeutically, mice were treated with 40 mg/kg of JIB-04 (MCE) (dissolved in 12.5% Cremophor EL, 12.5% DMSO, aqueous suspension) by intragastric gavage, starting 1 week after CCL4 injections, three time a week for 3 weeks. Mice were sacrificed 48 h after the final injection for subsequent analysis.

High-Fat Diet (HFD)-Induced Fibrosis: Mice aged 8 to 10 weeks were fed either a normal diet (ND) or high-fat diet (HFD) (PNBIO) for 16 weeks. For the HFD group, mice were also injected intraperitoneally with 2 ml/kg of CCL4 (diluted in a 1:9 ratio with corn oil) biweekly to better simulate non-alcoholic steatohepatitis (NASH) pathological characteristics (38). Mice were sacrificed 48 h after the final injection for subsequent analysis.

### Isolation of primary liver nonparenchymal cells (NPCs)

Hepatic NPCs were isolated as described previously (39). Briefly, 6 to 8 weeks mouse livers were perfused *in situ via* the portal vein with Hank’s solution containing 0.5 mg/ml collagenase IV (Gibco) and 10 μg/ml DNase I (Roche), and digested at 37 °C for 20 min. The suspension was filtered through a 70 μm mesh, centrifuged at 350*g* 4 °C for 10 min, and the nonparenchymal fraction was collected. Erythrocytes were lysed, and the NPCs were processed for further analysis. Kupffer cells (KCs) and hepatic stellate cells (HSCs) were separated by 70% and 30% Percoll density gradient centrifugation. KCs were cultured for 2 h to remove non-adherent cells. HSCs were cultured for 6 h to remove non-adherent cells.

### Induction of polarization and activation

KCs: Treated with LPS (100 ng/ml, Sigma) and IFN-γ (20 ng/ml, Peprotech) for 48 h to induce M1 polarization or with IL-4 (20 ng/ml, Peprotech) and IL-13 (20 ng/ml, Peprotech) for M2 polarization.

HSCs: Cultured *in vitro* for 6 days for spontaneous activation.

### Isolation of bone marrow-derived macrophages (BMDMs)

Following established protocols (40), femurs and tibiae were dissected. After flushing the bone marrow cavity, centrifugation was performed. After lysing erythrocytes, non-adherent cells were cultured with 10 ng/ml M-CSF (Peprotech) for 7 days to generate BMDMs.

### Flow cytometry analysis

Cells were collected, washed with PBS containing 1% FBS, and blocked with CD16/32 for 10 min. Samples were stained with fluorescence-conjugated antibodies in the dark for 30 min, washed, filtered through 300-mesh filters, and analyzed using a Beckman CytoFlexS flow cytometer. Data were processed with FlowJo software. Antibody details are provided in Table S2.

### Co-Immunoprecipitation (Co-IP) assays

Co-IP assays were performed using kits specific for Flag-tag (Beyotime, P2181S), HA-tag (Beyotime, P2185S), or Protein A+G (Beyotime, P2179S). Cell lysates were prepared in lysis buffer with protease inhibitors. Lysates (1 mg protein) were incubated with specific magnetic beads or Protein A+G beads conjugated to 2 μl IgG/antibodies overnight at 4 °C. After washing, bound proteins were eluted with SDS sample buffer and analyzed by western blotting. Antibody details are provided in Table S3.

### Chromatin Immunoprecipitation (ChIP) assays

ChIP assays were conducted using the ChIP Assay Kit (Beyotime, P2080S). Briefly, cells were fixed with formaldehyde (10 min). Cell pellets were solubilized in ChIP lysis buffer and followed by pulsed ultrasonication to shear cellular DNA (20% ampl, 60 s). After centrifugation, 1% by volume the supernatant was reserved as input. The remaining sample was divided into several aliquots and incubated with 3 μl IgG or specific antibodies overnight at 4 °C, followed by incubation with magnetic beads for 2 h qPCR was used to quantify DNA. The samples were standardized using the percent input method, where the signal of the ChIP sample was divided by the signal of the input sample. The IgG antibody group served as the negative control for the antibody groups. Primer and antibody details are provided in Tables S3 and S5.

### Immunohistochemistry (IHC)

IHC was performed as we previously reported (35, 36). Tissues were fixed in 4% paraformaldehyde for 48 h, embedded in paraffin, and sectioned at 4 μm thickness. Sections underwent antigen retrieval under high pressure and temperature for 8 min, followed by overnight incubation with primary antibodies at 4 °C. After incubating with the corresponding secondary antibody, the sections were developed using DAB (MXB, 2031). Antibody details are provided in Table S3.

### Cell counting Kit-8(CCK8)Detection

Added 10 μl of CCK-8 reagent (Vazyme, A311) to each well. Incubated the plate for 2 h in the incubator, and used an enzyme label reader (Varioskan Flash, Thermo) to measure the OD value at 450 nm.

### Real-time Quantitative PCR (RT-qPCR)

Total RNA was extracted from cells using trizol and reverse-transcribed into cDNA with a reverse transcription kit (Vazyme, R323). mRNA levels were quantified using SYBR Green Master Mix (Vazyme, Q712) with gene-specific primers (provided in Table S4).

### Western blotting

Protein lysates were prepared using RIPA buffer (Solarbio, R0020) containing PMSF (Solarbio, P0100). Proteins were denatured, separated *via* SDS-PAGE, and transferred onto PVDF membranes (Millipore, ISEQ00010). Membranes were incubated overnight with primary antibodies at 4 °C, followed by secondary antibody (1:10,000, HRP Goat Anti-Rabbit/Mouse IgG) incubation at room temperature for 1 h. Signals were visualized using ECL detection reagents (Thermo, 34580). Each blot was performed in triplicate. Antibody details are provided in Table S3.

### Cell culture

KCs and BMDMs were cultured in RPMI 1640 medium (BasalMedia), AMs, HSCs, 293T and LX2 cells were cultured in DMEM supplemented with 10% FBS (Cegrogen) and 1% antibiotics (Invitrogen) at 37 °C in a 5% CO2 incubator. All cell lines were tested for *mycoplasma* contamination (Beyotime) and used within 6 months.

### Induction of LX2 activation

LX2 cells were serum-starved in basal medium for 12 h before being treated with TGF-β1 (10 ng/ml, Millipore) or IL-6 (40 ng/ml, PeproTech) for 48 h.

### Construction of shRNA and overexpression plasmids

shRNA plasmids targeting *Men1* and *Setd2* were constructed using the pLKO.1 vector. Sequence information is detailed in Table S1.

Overexpression plasmids were constructed using the pLNCX2 vector. Target gene fragments were amplified *via* PCR and ligated into the vector.

Overexpression plasmids were directly transfected into 293T or LX2 cells using lipo3000 (Beyotime)/PEI (MCE) for transient overexpression.

### Construction of knockdown and overexpression cells

shRNA plasmids, along with packaging plasmids psPAX2 and pMD2.G, were transfected into 293T cells using PEI for viral production. Viral supernatants were collected after 48 h.

Cells were treated with control or shRNA viral supernatants for 24 h, followed by selection with 10 μg/ml puro for 48 h to establish stable knockdown cell lines.

Overexpression plasmids, along with packaging plasmid pVSVG, were transfected into GP2-293 cells using PEI for viral production. Viral supernatants were collected after 48 h.

Cells were treated with control or overexpression viral supernatants for 24 h and selected with 0.5 mg/ml G418 for 5 days to establish stable overexpression cell lines.

### Masson and Sirius Red Staining

Masson Staining: Performed using the Masson Trichrome Staining Kit (Servicebio, G1006) according to the manufacturer’s instructions.

Sirius Red Staining: Performed with saturated picric acid containing 0.1% Fast Green and Direct Red.

### Enzyme-linked immunosorbent assay

Serum levels of IL-6 and TNF-α were measured using ELISA kits (Solarbio, SEKM-0007 and SEKM-0034) according to the manufacturer’s protocols. The standard samples were measured, and a standard curve was generated through linear fitting. Each sample was measured twice, and the average value was taken. The corresponding concentration of the sample was then determined by referencing the standard curve.

### TUNEL staining

TUNEL staining was performed using the TMR (Red) TUNEL Cell Apoptosis Detection Kit (Servicebio, G1502) according to the manufacturer's protocol.

### Biochemical measurement

Serum ALT, AST, TG, and TC levels were measured using kits from Nanjing Jiancheng (ALT: C009-2-1; AST: C010-2-1; TG: A110-1-1; TC: A111-1-1) according to the manufacturer’s protocols.

### Isolation of alveolar macrophages (AMs)

Animals were euthanized, and their tracheas were cannulated. Lavage was performed with three aliquots of 0.8 ml of ice-cold PBS (pH 7.3) for the whole lung lavage. The lavage fluid was centrifuged, and non-adherent cells were removed after 2 h of culture.

### Public database analysis

Gene expression related to *Men1*, *Setd2*, and *Kmt2a* in liver fibrosis was analyzed using the GSE14323 database (41). Expression of *Men1* in different stages of cirrhosis was analyzed using the GSE84044 database (42). Mouse models of fibrosis (MCD: GSE35961 (43), CCL4: GSE141821 (44), BDL: GSE152494 (45)) were analyzed for the *Men1* expression. Expression correlations between *Men1*/*Kmt2a* and collagen genes in HCC samples were analyzed using the TCGA PanCancer Atlas database *via* cBioPortal.

### Quantification methods

All immunohistochemical staining indicators were statistically analyzed using ImageJ software. The specific method was as follows: for each sample, 12 fields of view were randomly selected under a 20× objective lens, and the following were measured: (1) the percentage of positive area for α-SMA (thresholding parameters: 180) and Sirius Red (thresholding parameters: 120) staining; (2) the count of F4/80 positive cells (Manual counting using the Cell Counter Plugin tool). The final statistical result for each sample was obtained by averaging the measurements from the 12 fields of view.

All Western blots were statistically analyzed using ImageJ software. The grayscale value of the target protein is ratioed with the grayscale value of the reference protein (β-actin) to obtain the standardized relative expression level of the protein.

### Statistical analysis

The data are presented as mean values ± standard error of mean (SEM), and individual data points (representing individual mice or biological replicates) are included in all graphs to demonstrate the distribution. Statistical significance was determined using GraphPad Prism9 software and unpaired *t*-tests were used. The figure legends provide details on the number of samples or mice per group and the replicates in independent experiments. For analyzing the correlation between *Men1/Kmt2a* and collagen-related genes, the Pearson correlation coefficient is used to calculate the *r*-value, and a *t* test is employed to compute the *p*-value.

## Data availability

All data are available in the main text or the supporting information. All data reported in this paper will be shared by the lead contact upon request. Any additional information required to reanalyze the data reported in this paper is available from the lead contact upon request.

## Supporting information

This article contains supporting information.

## Conflict of interest

The authors declare that they have no conflicts of interest with the contents of this article.
