# Supplementary material for: Myeloid loss of scaffolding protein menin promotes liver fibrosis via H3K36me3 reprogramming
Source: J Biol Chem. 2025 Jul 10;301(8):110471. doi: 10.1016/j.jbc.2025.110471 (PMC12346064; doi:10.1016/j.jbc.2025.110471)
Supplement: Figs. S1–S6 [file mmc1.docx]

**Myeloid** **loss of scaffold protein menin promotes liver fibrosis via H3K36me3 reprogramming**

Qing Han^1^, Yujun Chen^1^, Junbo Yuan^1^, Li Zhang^1^, Qifan Zheng^1^, Guanghui Jin^1^

Figure S1 to Figure S6

**
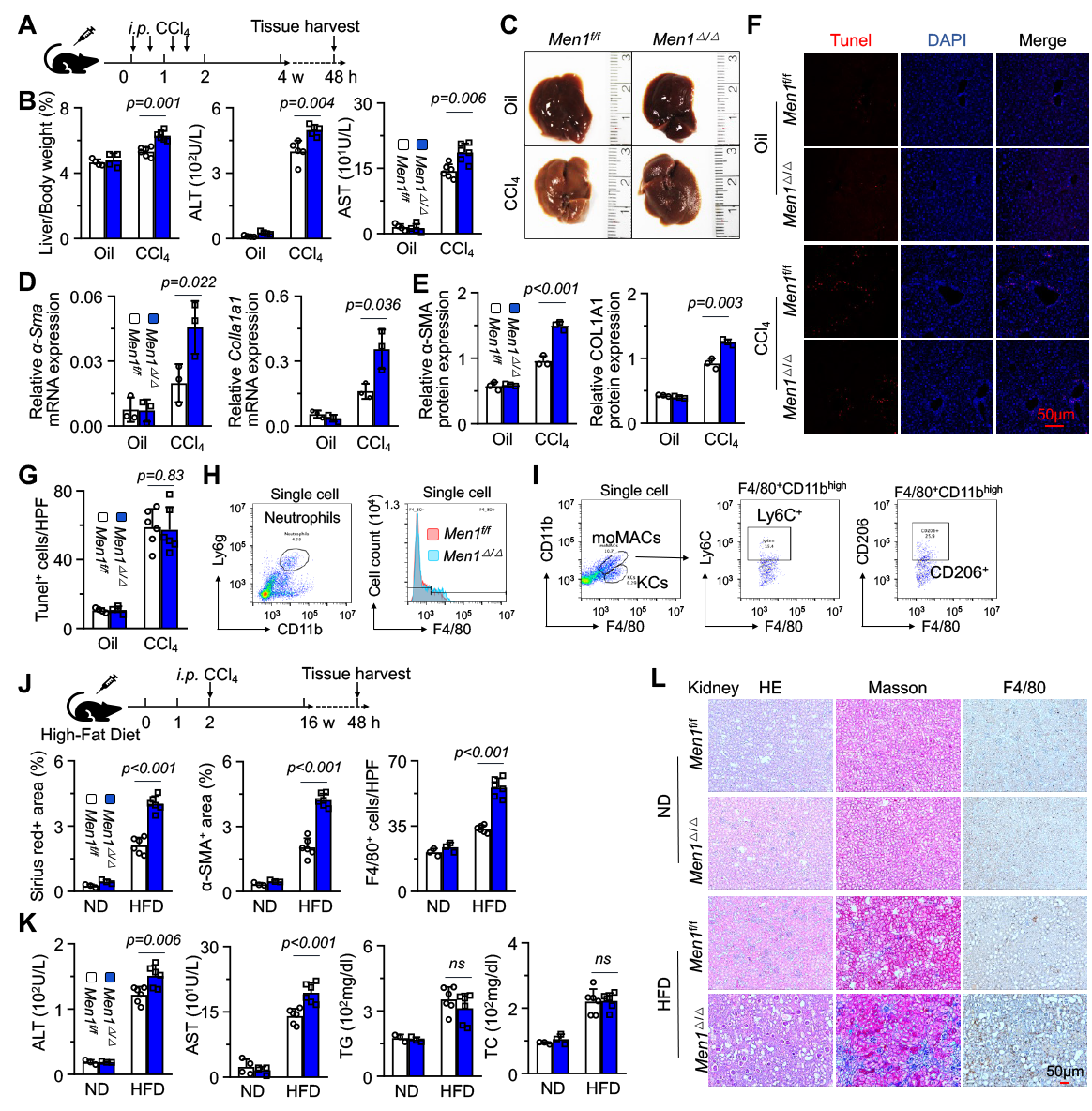
Figure S1. Loss of *Men1* promotes liver fibrosis.** (**A**) Schematic diagram for CCL4-induced liver fibrosis model. (**B**) Quantification of liver/body weight ratio, serum alanine aminotransferase (ALT), and aspartate aminotransferase (AST) levels of 1A (Oil n=4, CCL4 n=6). (**C**) Gross morphology of livers from above-mentioned models in 1A. (**D**) RT-qPCR analysis of *α-Sma* and *Colla1a1* mRNA expression in livers from above-mentioned models in 1A (Oil n=3, CCL4 n=3). (**E**) Relative quantification of protein expression levels (normalized to β-actin) of 1C (n=3). (**F** and **G**) Representative and quantification of TUNEL staining in livers of 1A (Scale bar = 50 μm) (Oil n=4, CCL4 n=6). (**H** and **I**) FCM sorting panel for NEUs, MACs, KCs, and moMACs (Fig. 1, D to F). (**J**) Schematic diagram of HFD-induced liver fibrosis model. Quantification of α-SMA, SR, and F4/80 staining of 1H (ND n=3, HFD n=6). (**K**) Quantification of serum ALT, AST, triglyceride (TG), and total cholesterol (TC) levels of 1H (ND n=3, HFD n=6). (**L**) Representative staining of kidney (ND and HFD) with HE, Masson, and F4/80 from above-mentioned models in 1H (Scale bar = 50 μm). Unpaired *t*-test. Data are presented as mean ± SEM. Individual data points as independent biological replicates.

**
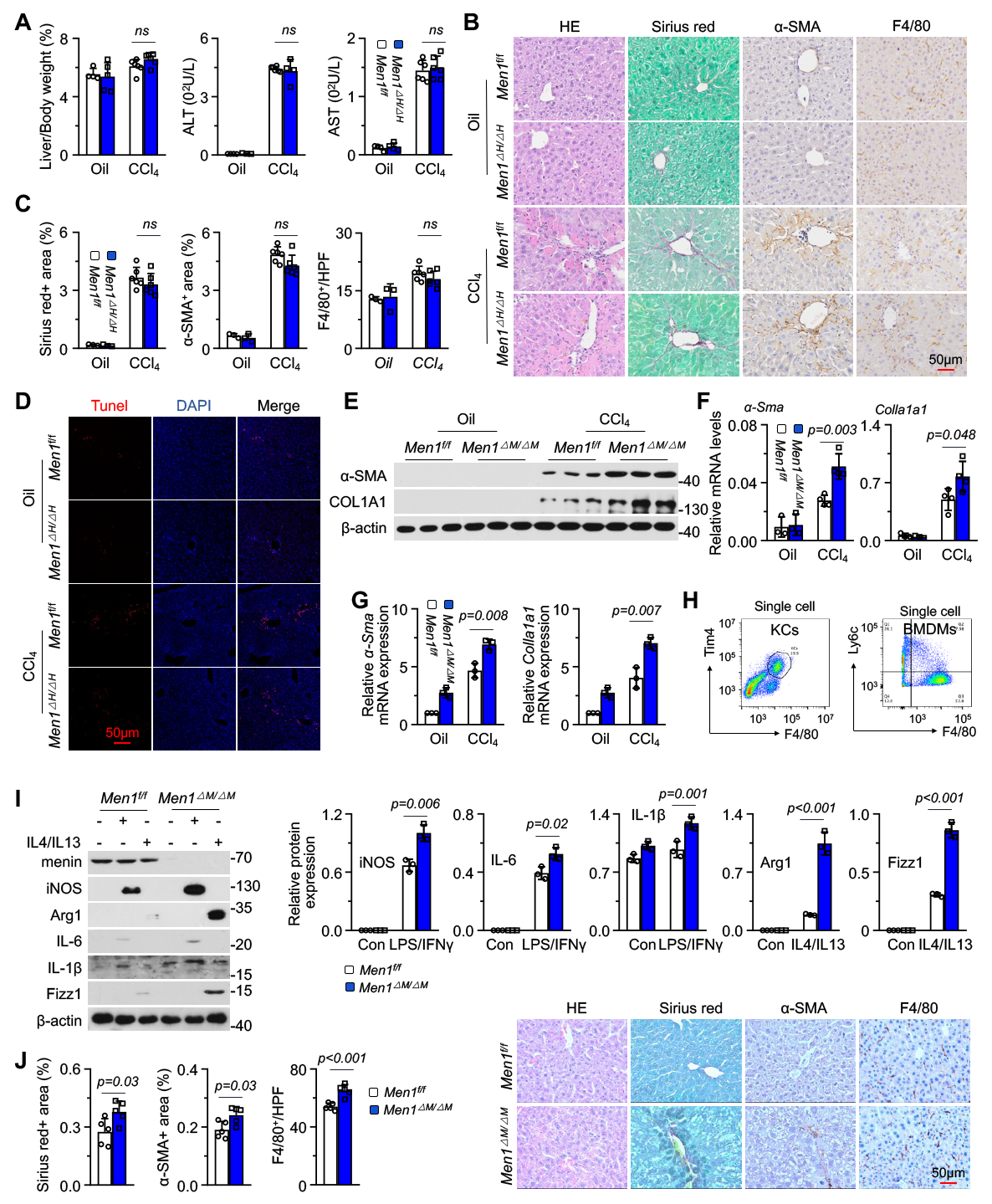
Figure S2.** **Myeloid deletion of *Men1* promotes liver fibrosis.** (**A** and **B**) Quantification of liver/body weight ratio, serum ALT, and AST levels for *Men1^f/f^* and *Men1^∆H/∆H^* mice treated with 2 ml/kg oil or CCL4 *i.p.*, twice a week for 4 weeks (Oil n=3-5, CCL4 n=6). Representative IHC staining of livers with HE, α-SMA, SR, and F4/80 (Scale bar = 50 μm). (**C**) Quantification of α-SMA, SR, and F4/80 staining (Oil n=3, CCL4 n=6) from above-mentioned models in S2A. (**D**) Representative TUNEL staining in liver tissues from above-mentioned models in S2A. (**E**) Western blot analysis of indicated protein expression in livers of 2A. (**F**) RT-qPCR analysis of *α-Sma* and *Colla1a1* mRNA expression in livers of 2A (Oil n=3, CCL4 n=4). (**G**) HSCs were isolated from above-mentioned models in 2A. RT-qPCR was used to detect the mRNA expression of *α-Sma* and *Colla1a1* (Oil n=3, CCL4 n=3). (**H**) FCM sorting panel for KCs in normal liver and BMDMs (Fig 2F). (**I**) The KCs isolated from *Men1^f/f^* and *Men1^∆M/∆M^* mice were treated with LPS/IFN-γ or IL-4/IL-13, respectively. The western blot was used to detect protein expressions of iNOS, Arg1, IL-1β, IL-6, Fizz1, and menin and the relative quantification of these protein expression levels (normalized to β-actin) (n=3). (**J**) Representative and quantification of HE, α-SMA, SR, and F4/80 staining in livers of *Men1^f/f^* and *Men1^∆M/∆M^* mice fed a chow diet for 8 months (Scale bar = 50 μm) (n=5). Unpaired *t*-test. Data are presented as mean ± SEM. Individual data points as independent biological replicates.

**
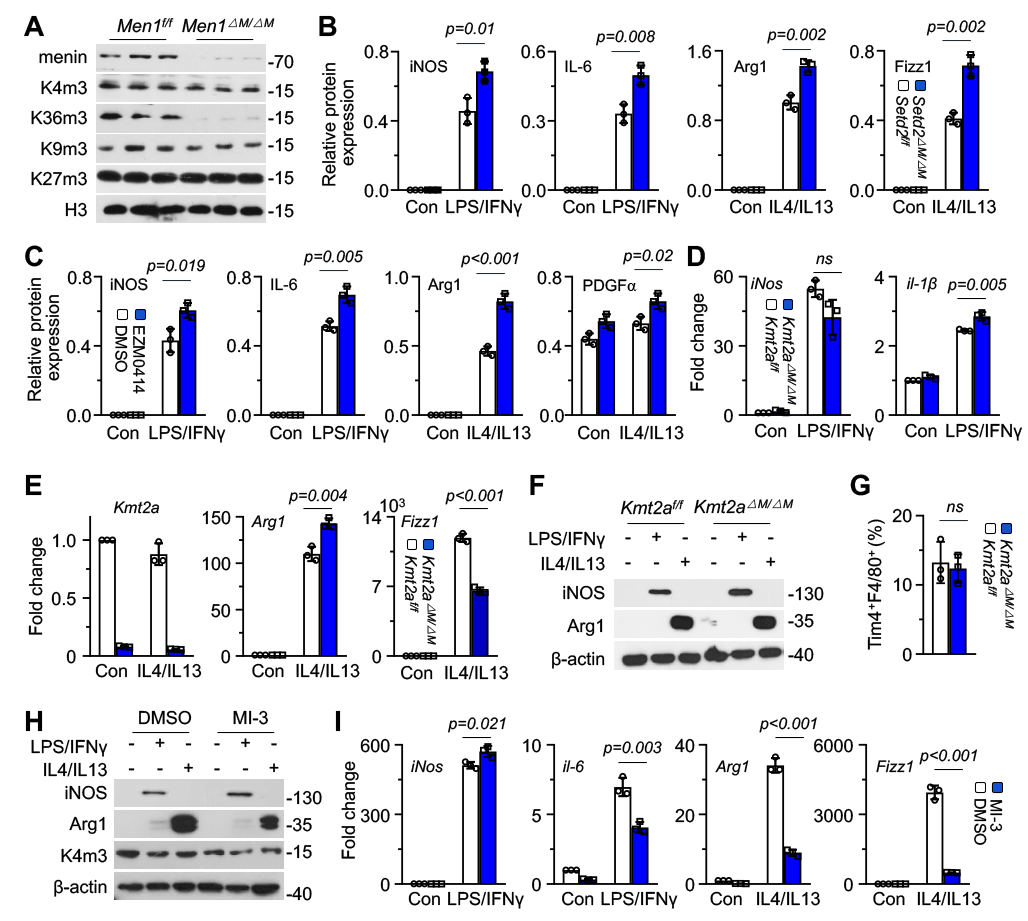
Figure S3. Menin-mediated regulation of KCs depends on SETD2.** (**A**) Western blot detection of indicated histone modifications in KCs isolated from 6-8 weeks *Men1^f/f^* and *Men1^∆M/∆M^* mice. (**B**) Relative quantification of protein expression levels (normalized to β-actin) of 3B (n=3). (**C**) Relative quantification of protein expression levels (normalized to β-actin) of 3G (n=3). (**D** to **F**) The KCs isolated from *Kmt2a^f/f^* and *Kmt2a^∆M/∆M^* mice were treated with LPS/IFNγ or IL4/IL13, respectively. RT-qPCR analysis of *Kmt2a*, *iNos*, *il-1β*, *Arg1*, and *Fizz1* mRNA expression (n=3) and western blotting detection of iNOS and Arg1 protein expression. (**G**) NPCs were isolated from liver of 6-8 weeks *Kmt2a^f/f^* and *Kmt2a^∆M/∆M^* mice, FCM was used to determine the proportion of Tim4^+^F4/80^+^ KCs (n=3). (**H** and **I**) The primary isolated KCs from WT-C57BL/6J mice were treated with LPS/IFNγ or IL4/IL13, and simultaneously administered MI-3 (10 μM) for 48 hours, respectively. RT-qPCR was used to detect *iNos*, *il-6*, *Arg1*, and *Fizz1* mRNA expression (n=3). Western blot was used to detect iNOS, Arg1, and H3K4me3 protein expression. Unpaired *t*-test. Data are presented as mean ± SEM. Individual data points as independent biological replicates.

**
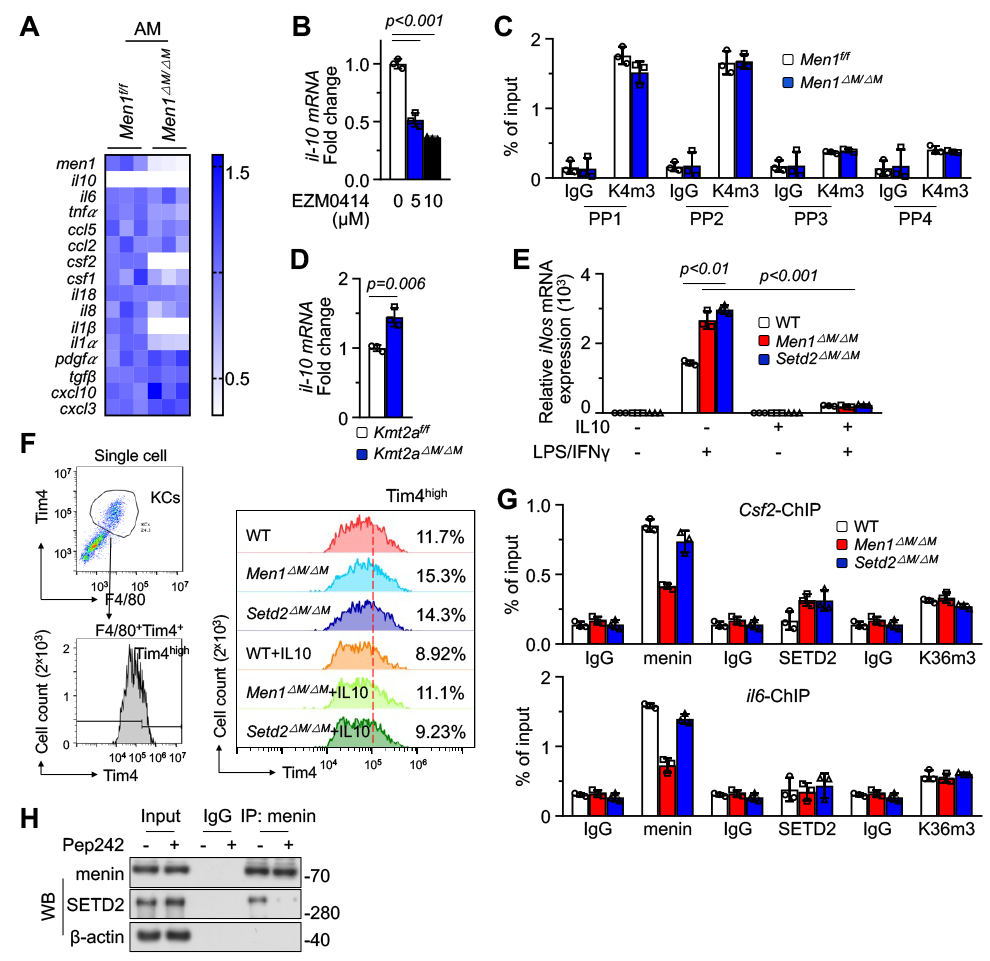
Figure S4. Menin/SETD2 controls KC remodeling through IL-10.** (**A**) RT-qPCR was used to analysis the mRNA expression of indicated genes in alveolar macrophages (AM) isolated from 6-8 weeks *Men1^f/f^* and *Men1^∆M/∆M^* mice (n=3). (**B**) The KCs isolated from 6-8 weeks WT-C57BL/6J mice were treated with EZM0414 for 48 hours, and RT-qPCR was used to detect *il-10* mRNA expression (n=3). (**C**) ChIP-qPCR analysis of enrichment of H3K4me3 on *il-10* regulatory region in KCs isolated from *Men1^f/f^* and *Men1^∆M/∆M^* mice (n=3). (**D**) RT-qPCR analysis of *il-10* mRNA expression in KCs isolated from 6-8 weeks *Kmt2a^f/f^* and *Kmt2a^∆M/∆M^* mice (n=3). (**E**) The KCs isolated from WT, *Men1^∆M/∆M^* and *Setd2^∆M/∆M^* mice were treated with LPS/IFNγ and simultaneously treated with IL-10 (50 ng/ml) for 48 hours. RT-qPCR analysis of *iNos* mRNA expression (n=3). (**F**) FCM sorting panel for Tim4^high^ KC subsets. (**G**) ChIP-qPCR analysis of enrichment of menin, SETD2, and H3K36me3 at *csf2* or *il-6* promoter in KCs isolated from WT, *Men1^∆M/∆M^* and *Setd2^∆M/∆M^* mice (n=3). (**H**) The KCs isolated from 6-8 weeks WT-C57BL/6J mice were treated with Pep242 (50 μg/ml) for 48 hours. Endogenous IP was performed with menin antibody and western blot was used to detect SETD2 and menin. Unpaired *t*-test. Data are presented as mean ± SEM. Individual data points as independent biological replicates.

**
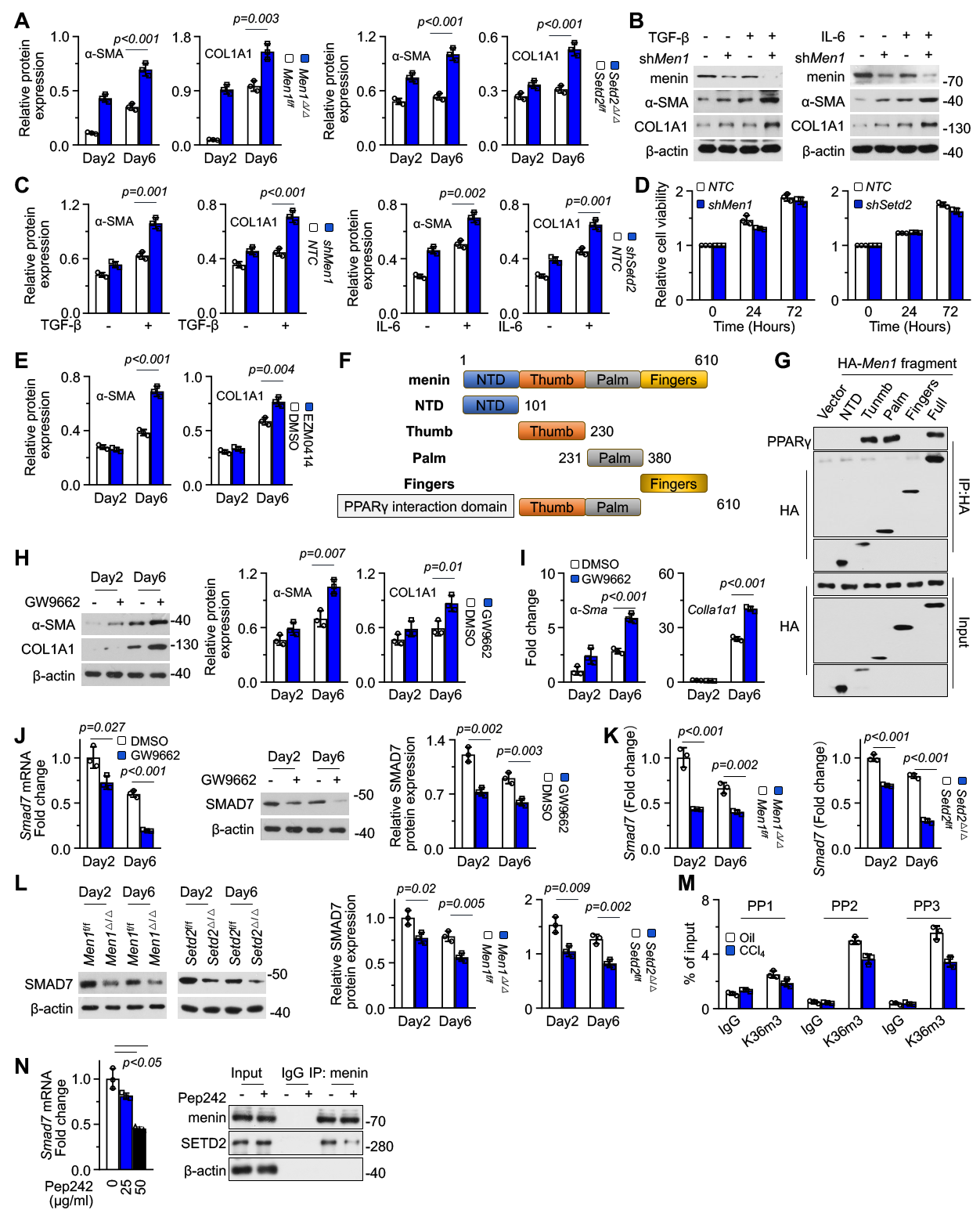
Figure S5. Menin/SETD2 collectively controls HSC activation.** (**A**) Relative quantification of protein expression levels (normalized to β-actin) of 5C and 5D (n=3). (**B**) The LX2 cells with *Men1* knockdown were treated with TGF-β (10 ng/ml) or IL-6 (40 ng/ml) for 48 hours. Western blot was used to detect menin, α-SMA, and COL1A1. (**C**) Relative quantification of protein expression levels (normalized to β-actin) of S5B (n=3). (**D**) The LX2 cells with *Men1/Setd2* knockdown were treated with TGF-β (10 ng/ml). CCK8 assay was used to detect the cell proliferation. (**E**) Relative quantification of protein expression levels (normalized to β-actin) of 5D (n=3). (**F**) Schematic of interaction domains between PPARγ and menin. (**G**) Co-IP was performed with anti-HA antibody labeled magnetic beads in 293T cells transfected with different fragments of HA-tagged menin. Western blot was used to detect HA and PPARγ. (**H**) The HSCs isolated from WT-C57BL/6J mice were cultured in *vitro* for 2 or 6 days and treated with GW9662 (10 μM). Western blot was used to detect α-SMA and COL1A1 and the relative quantification of these protein expression levels (normalized to β-actin) (n=3). (**I**) The HSCs isolated from WT-C57BL/6J mice were cultured in *vitro* for 2 or 6 days and treated with GW9662 (10 μM). RT-qPCR was used to detect *α-Sma* and *Colla1α1* expression (n=3). (**J**) RT-qPCR was used to detect *Smad7* expression in HSCs of S5H (n=3) (left), western blot was used to detect SMAD7 and the relative quantification of its protein expression level (normalized to β-actin) in HSCs of S5H (n=3) (right). (**K**) RT-qPCR (n=3) analysis of *Smad7* expression in HSCs of 5A and 5B (n=3). (**L**) Western blot analysis of SMAD7 expression in HSCs of 5A and 5B and the relative quantification of these protein expression levels (normalized to β-actin) (n=3). (**M**) ChIP-qPCR analysis of enrichment of H3K36me3 at *Smad7* regulatory region in HSCs isolated from 6-8 weeks WT-C57BL/6J mice treated with 2 ml/kg oil or CCL4 *i.p.*, twice/week for 3 weeks (n=3). (**N**) HSCs isolated from 6-8 weeks WT-C57BL/6J mice were treated with 50μg/ml Pep242 for 48 hours. RT-qPCR was used to detect *Smad7* mRNA expression (n=3) (left). Endogenous IP was performed with menin antibody and western blot was used to detect SETD2 and menin (right). Unpaired *t*-test. Data are presented as mean ± SEM. Individual data points as independent biological replicates.

**
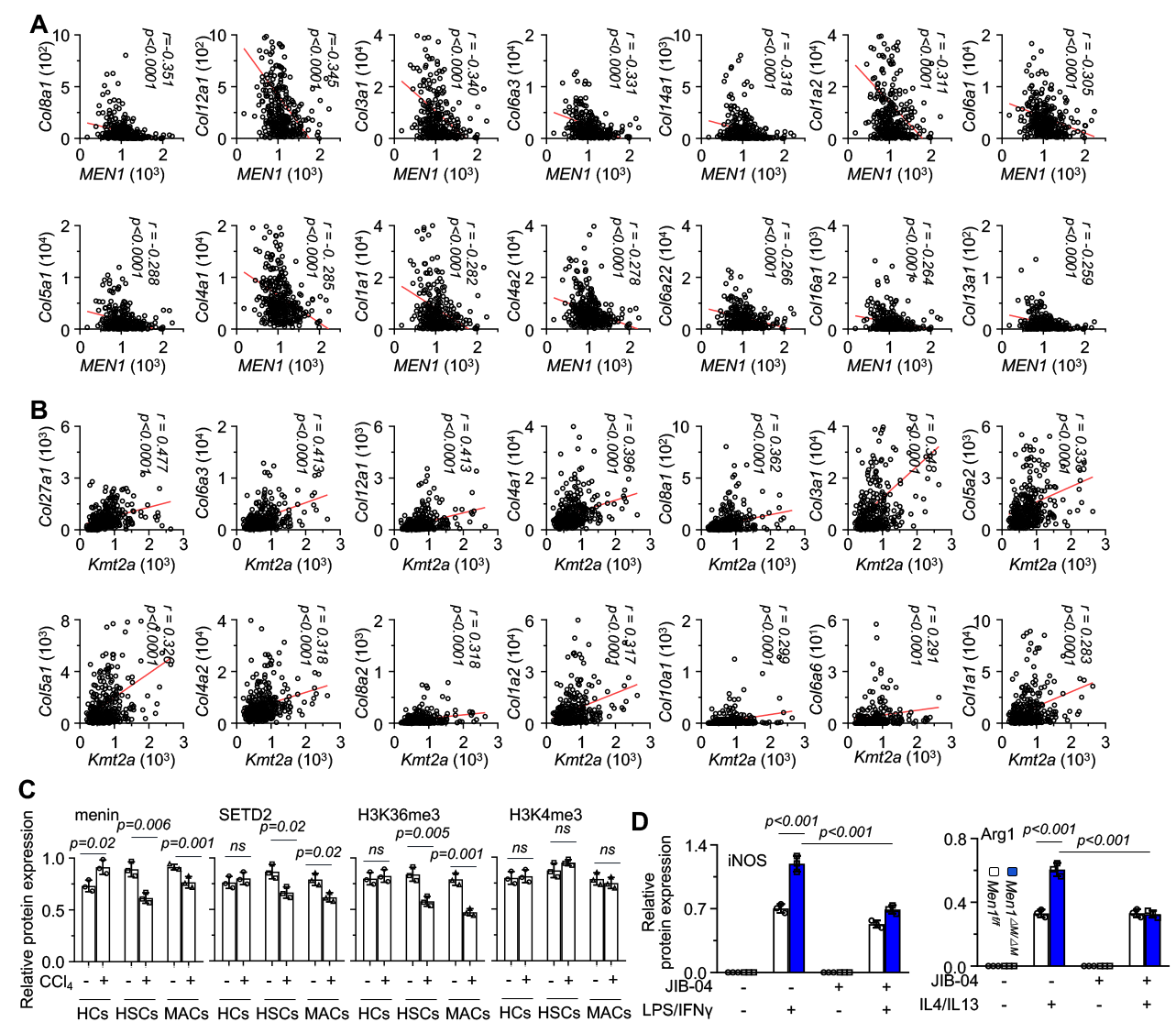
Figure S6. Therapeutic potential of targeting H3K36me3 reprogramming in liver fibrosis.** (**A** and **B**) TCGA databases analysis of correlations between *Men1*/*Kmt2a* and collagen-related genes mRNA expression in HCC samples (TCGA, PanCancer Atlas). (**C**) Relative quantification of protein expression levels (normalized to β-actin) of 6D (n=3). (**D**) Relative quantification of protein expression levels (normalized to β-actin) of 6F (n=3). Two-sided two-sample student’s *t*-test for (A and B), data are presented as normalized mRNA expression values (A and B). Unpaired *t*-test for (C and D), data are presented as mean ± SEM (C and D). Individual data points as independent biological replicates.
