# Supplementary material for: Myeloid loss of scaffolding protein menin promotes liver fibrosis via H3K36me3 reprogramming
Source: J Biol Chem. 2025 Jul 10;301(8):110471. doi: 10.1016/j.jbc.2025.110471 (PMC12346064; doi:10.1016/j.jbc.2025.110471)
Supplement: Tables S1–S6 [file mmc2.docx]

**Myeloid** **loss of scaffolding protein menin promotes liver fibrosis via H3K36me3 reprogramming**

Qing Han^1^, Yujun Chen^1^, Junbo Yuan^1^, Li Zhang^1^, Qifan Zheng^1^, Guanghui Jin^1^

Table S1 to Table S6

**Table S1. The list of shRNA used in this paper.**

| shRNA | sequence |
| --- | --- |
| sh*Men1* | CCATAATGAAACTGCCTCAA |
| sh*Setd2* | CCTGAAGAATGATGAGATAAT |

**Table S2. The list of antibodies used for flow cytometry analysis.**

| Antibodies | Catalog Number | Vendor |
| --- | --- | --- |
| CD11B-PE | 557397 | BD |
| LY6G | 127623 | Biolegend |
| F4/80 | 2555809 | eBioscience |
| CD11B-percpcy5.5 | 101228 | Biolegend |
| LY6C | 128006 | Biolegend |
| CD206 | 141706 | Biolegend |
| TIM4 | 130005 | Biolegend |
| CD16/32 | 156604 | Biolegend |

**Table S3. The list of antibodies used for Western blotting, IHC, and CHIP.**

| Antibodies | Vendor | Catalog Number | Dilution |
| --- | --- | --- | --- |
| α-SMA | abcam | ab5694 | 1:1000(western)  1:400(IHC) |
| F4/80 | Servicebio | GB11027 | 1:800(IHC) |
| menin | BETHYL | A300-105A | 1:4000(western) |
| β-actin | MultiSciencesBiotech | Mab1445 | 1:8000(western) |
| COL1A1 | CST | 72026 | 1:1000(western) |
| Ki67 | abcam | ab16667 | 1:400(IHC) |
| CD8 | CST | 98941 | 1:200(IHC) |
| SETD2 | CST | 80290 | 1:1000(western) |
| H3K36me3 | CST | 4909S | 1:1000(western)  1:500(IHC) |
| H3K4me3 | ABclonal | A22225 | 1:1000(western) |
| H3K9me3 | abcam | ab8898 | 1:1000(western) |
| H3K27me3 | abcam | ab4729 | 1:1000(western) |
| IgG | ABclonal | AC005 | 3µl/sample |
| PPARγ | ABclonal | A11183 | 1:1000(western) |
| Flag | CST | 14793 | 1:1000(western) |
| HA | abcam | ab18181 | 1:1000(western) |
| SMAD7 | abcam | ab216428 | 1:1000(western) |
| IL-6 | ABclonal | A22222 | 1:1000(western) |
| IL-1β | ABclonal | A22257 | 1:1000(western) |
| PDGFα | UpingBio | YP-Ab-15954 | 1:1000(western) |
| RETNα | abcam | ab39626 | 1:1000(western) |

**Table S4. The list of primers used for RT-qPCR.**

| Gene | Primers-Forward | Primers-Reverse |
| --- | --- | --- |
| m *iNos* | GGTGAAGGGACTGAGCTGTTA | CAACGTTCTCCGTTCTCTTGC |
| m *Arg1* | TTTTAGGGTTACGGCCGGTG | CCTCGAGGCTGTCCTTTTGA |
| m *α-Sma* | CGGGAGAAAATGACCCAGATT | GGACAGCACAGCCTGAATAGC |
| m *Colla1a1* | GCACGAGTCACACCGGAACT | AAGGGAGCCACATCGATGAT |
| m C*olla1a2* | CTACTGGTGAAACCTGCATCCA | GGGCGCGGCTGTATGAG |
| m *il-1β* | GGTGTGTGACGTTCCCATTA | ATTGAGGTGGAGAGCTTTCAG |
| m *il-6* | CAAAGCCAGAGTCCTTCAGAG | GTCCTTAGCCACTCCTTCTG |
| m *Fizz1* | CCCTTCTCATCTGCATCTCC | CTGGATTGGCAAGAAGTTCC |
| m *Men1* | ACTGCCACTGTTATCCAAGAC | CGCTCTTCGCCTGTTTCTAG |
| m *Setd2* | CCAATGCTAACGCCACCGAGAG | GCCTTGTTCTGCTCCTCATAGTCC |
| m *Kmt2a* | TCTCAAACAGACTGACCAGC | CATCAGGAAACACAGCTCGT |
| m *Pdgfα* | GAGGAAGCCGAGATACCCC | TGCTGTGGATCTGACTTCGAG |
| m *Tgf-β* | TGCTAATGGTGGACCGCAA | CACTGCTTCCCGAATGTCTGA |
| m *il-10* | AGTGGAGCAGGTGAAGAGTG | TTCGGAGAGAGGTACAAACG |
| m *Tnf-α* | CTTCTGTCTACTGAACTTCGGG | CAGGCTTGTCACTCGAATTTTG |
| m *ccl5* | CAAGTGCTCCAATCTTGCAG | ACCCTCTATCCTAGCTCATCTC |
| m *ccl2* | GTCCCTGTCATGCTTCTGG | GCTCTCCAGCCTACTCATTG |
| m *csf1* | AACAGCTTTGCTAAGTGCTCTA | ACTTCCACTTGTAGAACAGGAG |
| m *csf2* | GGCCTTGGAAGCATGTAGAGG | GGAGAACTCGTTAGAGACGACTT |
| m *il-18* | GACTCTTGCGTCAACTTCAAGG | CAGGCTGTCTTTTGTCAACGA |
| m *il-8* | GTCCTTAACCTAGGCATCTTCG | TCTGTTGCAGTAAATGGTCTCG |
| m *il-1α* | TGCAGTCCATAACCCATGATC | ACAAACTTCTGCCTGACGAG |
| m *cxcl10* | TCAGCACCATGAACCCAAG | CTATGGCCCTCATTCTCACTG |
| m *cxcl3* | CCCCAGGCTTCAGATAATCAT | AAAGACACATCCAGACACCG |
| m *Smad7* | GGCCGGATCTCAGGCATTC | TTGGGTATCTGGAGTAAGGAGG |
| m *actin* | TGTGATGGTGGGAATGGGTCAGAA | TGTGGTGCCAGATCTTCTCCATGT |
| m *Ym1* | TCTGGGTACAAGATCCCTGAA | TTTCTCCAGTGTAGCCATCCTT |

**Table S5. The list of primers used for CHIP-qPCR.**

| Gene | Primers-Forward | Primers-Reverse |
| --- | --- | --- |
| *il10*-pp1 | AACGAAGATCCTCCCCCGTA | GCTACACGTCCTGTTGACCA |
| *il10*-pp2 | TGCTGCCTGCTCTTACTGAC | CTAGGAGCATGTGGCTCTGG |
| *il10*-pp3 | GTGGGAGTGACTTTGAGGCA | TTCCCATGGAGAGAGAGGGG |
| *il10*-pp4 | GCAAGGGTGTCTCCTTCCTC | GCTCCACTGCCTTGCTCTTA |
| *smad7*-pp1 | CGCCTGCTGCCCCAACTCGGCG | GTCGTTTGCCGGCTAAGGAGCG |
| *smad7*-pp2 | AAGAAACTCAAGGAGCGGCA | GCCACCTGAACACTTTGCAC |
| *smad7*-pp3 | CAGTCCTAGGGCCCATCCCT | AGGACTTGTCCCTGCGGCC |
| *csf2*-pp1 | AGGCTGTCTGATGCTATTGC | CAGGATTCTAAGGGCTTGGG |
| *il6*-pp1 | CGGAGCTATTGAGACTGTGAG | GCGTGGAGAAAAGGGAAAAC |

**Table S6. The list of the referenced datasets.**

| author name | dataset title | data repository | year | global persistent identifier |
| --- | --- | --- | --- | --- |
| [Valeria R Mas](https://pubmed.ncbi.nlm.nih.gov/?term=Mas+VR&cauthor_id=19098997) | RMA expression data for liver samples from subjects with HCV, HCV-HCC, or normal liver | NCBI | 2009 | GSE14323 |
| [Mingjie Wang](https://pubmed.ncbi.nlm.nih.gov/?term=Wang+M&cauthor_id=28262670) | Characterization of gene expression profile in HBV-related liver fibrosis patients | NCBI | 2016 | GSE84044 |
| [Yuki Kita](https://pubmed.ncbi.nlm.nih.gov/?term=Kita+Y&cauthor_id=23028442) | Expression data from mouse liver treated with metformin | NCBI | 2012 | GSE35961 |
| [Philippe Lefebvre](https://pubmed.ncbi.nlm.nih.gov/?term=Lefebvre+P&cauthor_id=28679947) | Transcriptomic analysis of CLL4-induced liver injury in WT and DPT KO mice | NCBI | 2019 | GSE141821 |
| [Eva Gijbel](https://pubmed.ncbi.nlm.nih.gov/?term=Gijbels+E&cauthor_id=32152650)s | Robustness testing and optimization of an adverse outcome pathway on cholestatic liver injury | NCBI | 2020 | GSE152494 |
